# Two common and problematic leucochrysine species – *Leucochrysa (Leucochrysa) varia* (Schneider) and *L. (L.) pretiosa* (Banks) (Neuroptera, Chrysopidae): redescriptions and synonymies

**DOI:** 10.3897/zookeys.310.5071

**Published:** 2013-06-19

**Authors:** Catherine A. Tauber, Francisco Sosa, Gilberto S. Albuquerque

**Affiliations:** 1 Department of Entomology, Comstock Hall, Cornell University, Ithaca, NY 14853-2601 and Department of Entomology, University of California, Davis, CA, USA; 2 Museo Entomológico “Dr. José Manuel Osorio” (UCOB), Universidad Centroccidental “Lisandro Alvarado”, Venezuela; 3 Laboratório de Entomologia e Fitopatologia, CCTA, Universidade Estadual do Norte Fluminense, Campos dos Goytacazes, RJ, Brazil 28013-602

**Keywords:** Chrysopinae, Leucochrysini, *Leucochrysa*, *ampla*, *angrandi*, *delicata*, *erminea*, *internata*, *phaeocephala*, *variata*, *vegana*, *walkerina*

## Abstract

*We dedicate this article to the memory of Sergio de Freitas, FCAV-UNESP, Jaboticabal, São Paulo, Brazil (deceased, 2012). He was an active and enthusiastic Neuropterist and the cherished mentor and friend of Francisco Sosa*.

*Leucochrysa* McLachlan is the largest genus in the Chrysopidae, yet it has received relatively little taxonomic attention. We treat two problematic and common *Leucochrysa* species – *Leucochrysa (Leucochrysa) varia* (Schneider, 1851) and *Leucochrysa (Leucochrysa) pretiosa* (Banks, 1910). Both are highly variable in coloration and were described before the systematic importance of chrysopid genitalia was recognized. Recent studies show that these species occur within a large complex of cryptic species and that they have accumulated a number of taxonomic problems. We identify new synonymies for each of the species–for *Leucochrysa (Leucochrysa) varia*: *Leucochrysa (Leucochrysa) ampla* (Walker, 1853), *Leucochrysa internata* (Walker, 1853), and *Leucochrysa (Leucochrysa) walkerina* Navás, 1913; for *Leucochrysa (Leucochrysa) pretiosa*: *Leucochrysa (Leucochrysa) erminea* Banks, 1946. The synonymy of *Leucochrysa delicata* Navás, 1925 with *Leucochrysa (Leucochrysa) pretiosa* is stabilized by the designation of a neotype. The following species, which were previously synonymized with *Leucochrysa (Leucochrysa) varia* or *Leucochrysa (Leucochrysa) pretiosa*, are reinstated as valid: *Leucochrysa (Leucochrysa) phaeocephala* Navás, 1929, *Leucochrysa (Leucochrysa) angrandi* (Navás, 1911), and *Leucochrysa (Leucochrysa) variata* (Navás, 1913). To help stabilize *Leucochrysa* taxonomy, lectotypes are designated for *Allochrysa pretiosa* and *Allochrysa variata*. Finally, *Leucochrysa vegana* Navás, 1917 is considered a *nomen dubium*.

## Abstract (es)

*Leucochrysa* McLachlan es el género más grande de Chrysopidae, sin embargo, ha recibido poca atención taxonómica. Dos de las especies más comunes y problemáticas de *Leucochrysa* son estudiadas – *Leucochrysa (Leucochrysa) varia* (Schneider, 1851) y *Leucochrysa (Leucochrysa) pretiosa* (Banks, 1910). Ambas son altamente variables en coloración y fueron descritas antes de que se reconociera la importancia de la genitalia en la sistemática de Chrysopidae. Estudios recientes demuestran que estas especies pertenecen a un complejo de especies crípticas, razón por la cual han acumulado numerosos problemas taxonómicos. En este estudio se identificaron las siguientes sinonimias; para *Leucochrysa (Leucochrysa) varia*: *Leucochrysa (Leucochrysa) ampla* (Walker, 1853), *Leucochrysa internata* (Walker, 1853), y *Leucochrysa (Leucochrysa) walkerina* Navás, 1913; para *Leucochrysa (Leucochrysa) pretiosa*: *Leucochrysa (Leucochrysa) erminea* Banks, 1946. La sinonimia de *Leucochrysa delicata* Navás, 1925 con *Leucochrysa (Leucochrysa) pretiosa* es estabilizada por la designación de un neotipo. Las especies *Leucochrysa (Leucochrysa) phaeocephala* Navás, 1929, *Leucochrysa (Leucochrysa) angrandi* (Navás, 1911) y *Leucochrysa (Leucochrysa) variata* (Navás, 1913) las cuales fueron previamente sinonimizadas con *Leucochrysa (Leucochrysa) varia* o *Leucochrysa (Leucochrysa) pretiosa*, son reestablecidas como válidas. Para ayudar a estabilizar la taxonomía de *Leucochrysa*, se designaron lectotipos para *Allochrysa pretiosa* y *Allochrysa variata*. Finalmente, *Leucochrysa vegana* Navás, 1917 es considerada como un *nomen dubium*.

## Abstract (pt)

*Leucochrysa* McLachlan é o maior gênero de Chrysopidae; no entanto, ele tem recebido relativamente pouca atenção taxonômica. Nesse trabalho, duas espécies de *Leucochrysa* problemáticas e comuns são analisadas - *Leucochrysa (Leucochrysa) varia* (Schneider, 1851) e *Leucochrysa (Leucochrysa) pretiosa* (Banks, 1910). Ambas foram descritas antes da importância sistemática da genitália de crisopídeos ter sido reconhecida e são altamente variáveis na coloração. Estudos recentes têm demonstrado que essas espécies ocorrem dentro de um grande complexo de espécies crípticas e que elas têm acumulado diversos problemas taxonômicos. Foram identificadas novas sinonímias para cada uma das espécies - para *Leucochrysa (Leucochrysa) varia*: *Leucochrysa (Leucochrysa) ampla* (Walker, 1853), *Leucochrysa internata* (Walker, 1853) e *Leucochrysa (Leucochrysa) walkerina* Navás, 1913; para *Leucochrysa (Leucochrysa) pretiosa*: *Leucochrysa (Leucochrysa) erminea* Banks, 1946. A sinonímia de *Leucochrysa delicata* Navás, 1925 com *Leucochrysa (Leucochrysa) pretiosa* é estabilizada pela designação de um neótipo. As seguintes espécies, que eram previamente sinonimizadas com *Leucochrysa (Leucochrysa) varia* ou *Leucochrysa (Leucochrysa) pretiosa*, são reintegradas como válidas: *Leucochrysa (Leucochrysa) phaeocephala* Navás, 1929, *Leucochrysa (Leucochrysa) angrandi* (Navás, 1911) e *Leucochrysa (Leucochrysa) variata* (Navás, 1913). Para auxiliar na estabilização da taxonomia de *Leucochrysa*, lectótipos são designados para *Allochrysa pretiosa* e *Allochrysa variata*. Finalmente, *Leucochrysa vegana* Navás, 1917 é considerada *nomen dubium*.

## Introduction

*Leucochrysa* McLachlan is the largest genus in the family Chrysopidae; it is restricted to the New World, and it is most diverse and abundant in the Neotropics (Adams and Penny 1986). Currently, *Leucochrysa* includes two subgenera, *Leucochrysa (Leucochrysa)*, with ca 46 species, and *Leucochrysa (Nodita)*, with ca 150 species (Brooks and Barnard 1990, Penny 1998, 2001, Freitas and Penny 2001, Freitas 2005, Tauber et al. 2008, 2011a, 2011b). The genus has numerous taxonomic complications, many stemming from the poor status of its descriptive systematics. Most *Leucochrysa* species were described without reference to their internal anatomy (e.g., genitalia), and in many cases, these structures continue to remain unknown. As a result, identifications are difficult and a large number of cryptic species have gone unrecognized. In addition, polymorphisms and other forms of intraspecific variation are often interpreted as species differences; in some cases, males and females of the same species are described under different names. Moreover, systematic study of the group has been slow because types are sometimes difficult to locate or access; many are in poor condition. As a result, synonymies are numerous; species are difficult to identify with accuracy; a significant number of species remain undescribed; and the genus is a taxonomic enigma for systematists and ecologists who are interested in chrysopids.

Efforts to improve the descriptive systematics of *Leucochrysa* began in the late 1970s with work by Adams (1977, 1979); his studies were the first to include the genitalia (male and female), and his findings were strongly rooted in careful examination of type specimens. Subsequently, in the last ca 10 years, interest in the descriptive systematics of *Leucochrysa* has increased. Regional treatments of *Leucochrysa* have been published; new species have been described; polymorphisms have been elucidated; and, larval morphology has been explored (Freitas and Penny 2001, Penny 2002, Tauber 2004, Freitas 2005, Mantoanelli et al. 2006, 2011, Tauber et al. 2008, 2011a, 2011b). Unfortunately, in some of these studies, type specimens do not appear to have received appropriate attention. As a result, errors continue to creep into the literature on the taxonomy of the group.

Recently we examined specimens of *Leucochrysa (Leucochrysa)* that resemble the relatively common species, *Leucochrysa (Leucochrysa) varia* (Schneider). We were surprised to find that our specimens included numerous cryptic species. Among them was *Leucochrysa (Leucochrysa) pretiosa* (Banks); most, if not all, of the other species appear to be undescribed. To help provide a sound morphological and systematic basis for future comparative study and description of the newly discovered species, we examine the taxonomy and morphology of the two previously named species. In doing so, we (a) redescribe and provide images of the two species, including the genitalia of both males and females, (b) identify new synonymies for each of the species, (c) help stabilize the nomenclature of the group by designating a neotype and two lectotypes, (d) reinstate to valid status three species that had previously been synonymized with one or the other of the species, and (e) designate one species as a *nomen dubium*. All of these taxonomic actions are verified with images of the appropriate types.

## Materials and methods

We searched for specimens in a large number of entomological collections. Below are the institutions (with acronyms) where we found and used specimens.

Tauber: AMNH, American Museum of Natural History, New York, NY; CAS, California Academy of Sciences, San Francisco, CA; FMNH, Field Museum of Natural History, Chicago, IL; SEM, Snow Entomological Museum, University of Kansas, Lawrence, KS; LACM, Los Angeles County Museum of Natural History, Los Angeles, CA; ROM, Royal Ontario Museum, Toronto, ON, Canada; UCB, Essig Museum, University of California, Berkeley, CA; UID, W. F. Barr Entomological Collection, University of Idaho, Moscow, ID; USNM, National Museum of Natural History (formerly United States National Museum), Smithsonian Institution, Washington, D.C.; USU, Entomological Museum, Utah State University, Logan, UT.

Tauber and Albuquerque: BMNH, The Natural History Museum (formerly British Museum of Natural History), London, England; MCZ, Museum of Comparative Zoology, Harvard University, Cambridge, MA; MNHN, Muséum national d’Histoire naturelle, Paris, France.

Sosa: UCOB, Museo Entomológico “Dr. José Manuel Osorio”, Universidad Centroccidental “Lisandro Alvarado”, Barquisimeto, Lara, Venezuela; MIZA, Museum of the Institute of Agricultural Zoology, Universidad Central de Venezuela, Maracay, Aragua, Venezuela; FCAV-UNESP, Faculdade de Ciências Agrárias e Veterinárias, Universidade Estadual Paulista “Júlio de Mesquita Filho”, Jaboticabal, São Paulo, Brazil (Sergio de Freitas Collection).

Albuquerque: UENF, Insect Collection, Universidade Estadual do Norte Fluminense, Campos dos Goytacazes, Rio de Janeiro, Brazil.

For the genitalia studies, the entire abdomen or the apical region of the abdomen was removed and cleared in 10% KOH, tinged with Clorazol Black E, and transferred to glycerine. Subsequently, the structures were stored in plastic microvials attached to the corresponding specimens. Measurements were made with the aid of Image J 1.46 (NIH, public domain) (Tauber) and Motic Image Plus (version 2.0) (Sosa). All measurements were made as previously described (Tauber 2010); the numbers of specimens measured ranged from four (internal structures) to ten (external structures). We made frequent use of the Neuropterida website: http://lacewing.tamu.edu/index.html (Oswald 2006, 2007) and the images of *Leucochrysa* types in the MCZ On-line Type Database: http://insects.oeb.harvard.edu/MCZ/index.htm (all of which were captured for the MCZ by CAT, GSA and MJT in 2010).

## Shared characteristics of *Leucochrysa (Leucochrysa) varia*-like species

The two species that we are considering here are among a relatively large number of mostly undescribed *Leucochrysa (Leucochrysa)* species–all of which resemble each other very closely. We refer loosely to these species as “*varia*-like”. The species in this group generally express the following suite of external features: (1) head yellow to cream-colored, tinged slightly to extensively with red to red-wine coloration; (2) occiput tinged with red; (3) vertex raised, tinged with red laterally, with reddish brown transverse, V-shaped bar above antennae; (4) antenna with basal membrane variably tinged with red; (5) scape with red to red-wine coloration throughout; (6) pedicel generally cream-colored, with inner margin shaded brown; (7) flagellum cream-colored to yellow, covered with pale bristles, basal three to five flagellomeres generally with inner margin streaked with black; (8) frons and clypeus white to cream-colored or variably red suffused; (9) gena tinged with pink, red or reddish brown; (10) labrum yellowish, generally not marked; (11) maxillary and labial palpi yellowish; (12) cervix with small, red, lateral mark; (13) pronotum greenish to yellowish, unmarked; (14) mesonotum, metanotum variably pigmented with red, reddish brown or black (pigmentation with wide range of intraspecific and interspecific variation); (15) abdominal tergites 5-6 with dark brown oval spot, bordered with red and yellow.

Internal features, including any genital characters that may characterize this group will be considered in a later publication.

### Leucochrysa (Leucochrysa) varia

(Schneider, 1851)

http://species-id.net/wiki/Leucochrysa_varia

Figs 1 14

Chrysopa varia Schneider [1851] (1851: 154, Plate 58) original description: “Brasilia, ubi a Dr. Clausen inventa; (collect. E. de Selys-Longchamps!)”. Walker (1853: 268) brief redescription, collection records; McLachlan (1868a: 270) taxonomic note; Brooks and Barnard (1990: 247) confirmed type species of genus *Leucochrysa*.
Leucochrysa varia (Schneider). McLachlan (1868b: 208) first reference to combination, *Leucochrysa varia* designated as type species of genus; Navás (1917: 279) species list; Navás (1922a: 392) collection record; Navás (1922b: 89) collection record; Navás (1924: 28) collection record; Navás (1926: 12) collection record; Navás (1928a: 131) collection record (probably in error); Navás (1928b: 111) collection record; Navás (1928c: 35) collection record; Navás (1929: 862) collection records; Navás (1932: 57) collection record; Banks (1944: 31) note on type, species distribution records (probably in error); Banks (1945: 168) note on geographic distribution, comparison with *Leucochrysa pretiosa*; Penny (1977: 23) species list; Adams (1979: 97) probable occurrence in Mexico.
Allochrysa varia (Schneider). Banks (1910: 150) first reference to combination; Navás (1912-1913: 313) species list, collection record; Navás (1913: 156) brief redescription; Navás (1917: 279) genus synonymized with *Leucochrysa*.
Leucochrysa (Leucochrysa) varia (Schneider). Brooks and Barnard (1990: 248, 277, figs 498-507) subgeneric determination, species list, figures; Freitas and Penny (2001: 282, 354, fig. 43) brief redescription, collection records, figures -- based on misidentified specimens in the FCAV-UNESP, not *Leucochrysa (Leucochrysa) varia*; Mantoanelli et al. (2006: 7) description of larvae, developmental data, analysis of color polymorphism; Mantoanelli and Albuquerque (2007: 302) behavioral and developmental data; Oswald (2007) catalog listing.
Chrysopa internata Walker [1853] (1853: 252) original description: “*a*. Brazil. *b*. ----------? From Mr. Children’s collection”. McLachlan (1868a: 269) synonymy with *Chrysopa ampla* Walker.
Leucochrysa internata (Walker). Navás (1912-1913: 303) first reference to combination, species list; Navás (1913: 102) brief redescription, reversal of McLachlan’s 1868a synonymy with *Leucochrysa ampla*; Kimmins (1940: 444) identification of Walker’s specimen *b*, without locality data, as primary type; Penny (1977: 23) species list; Brooks and Barnard (1990: 277) apparently listed in error as a synonym of *Leucochrysa (Leucochrysa) walkerina* Navás; Oswald (2007) catalog listing as a synonym of *Leucochrysa (Leucochrysa) walkerina* as per Brooks and Barnard (1990). **Syn. n.**
Allochrysa internata (Walker). Banks (1914-1915: 625) first reference to combination; Navás (1917: 279, *internata* not specifically mentioned) genus synonymized with *Leucochrysa*; Oswald (2007) catalog listing as synonym of *Leucochrysa (Leucochrysa) walkerina*, apparently in error.
Nodita internata (Walker). Navás (1917: 280) first reference to combination, species list (probably an error); Oswald (2007) catalog listing as synonym of *Leucochrysa (Leucochrysa) walkerina*.
Chrysopa ampla Walker [1853] (1853: 268) original description: “*a*. Georgia. From Mr. Abbot’s collection. *b*. -------? From Mr. Children’s collection”. McLachlan (1868a: 270) taxonomic note and mention of close relationship with *Chrysopa varia*. Kimmins (1940: 444) identification of Walker’s specimen *b*, without locality data, as primary type. Penny (1977: 23) listing as a synonym of *Leucochrysa internata* (Walker), without comment.
Leucochrysa (Leucochrysa) ampla (Walker). Brooks and Barnard (1990: 276) first reference to generic, subgeneric combinations, species list; Freitas and Penny (2001: 280, 351, fig. 40) brief redescription, collection records, figures; Oswald (2007) catalog listing. **Syn. n.**
Leucochrysa vegana Navás [1917] (1917: 278) (not *Nodita vegana* Navás, 1925) original description: “Colombia: La Vega, Juni 1915 (Coll. Br. Apolinar Maria)”. Banks (1944: 30) synonymy with *Leucochrysa varia*. Penny (1977: 23) listing as a synonym; Brooks and Barnard (1990: 277) listing as a synonym; Oswald (2007) catalog listing as a synonym. **Nom. d.**
Leucochrysa phaeocephala Navás [1929] (1929b: 21) original description: “America: «Niederl. Guayan. Obere Commaryne, 28. 11. 1908». Mus. Hamburg”. Banks (1944: 30) synonymy with *Leucochrysa varia*. Penny (1977: 23) listing as a synonym; Brooks and Barnard (1990: 277) listing as a synonym; Oswald (2007) catalog listing as a synonym.
Leucochrysa (Leucochrysa) phaeocephala Navás. **Valid status reinstated**. See below.
Leucochrysa walkerina Navás [1913] (1913: 102) original description: “Brazil”. Penny (1977: 23), as “*Leucochrysa walkerina*Navás (1917)”, listing as a synonym of *Leucochrysa internata*, without comment.
Leucochrysa (Leucochrysa) walkerina Navás. Brooks and Barnard (1990: 277) subgeneric determination, species list; Freitas and Penny (2001: 283, 355, fig. 44) brief redescription, collection records, figures -- based on misidentified specimens in the FCAV-UNESP; Oswald (2007) catalog listing. **Syn. n.**

#### Known geographical distribution.

Our findings [based on confirmed published records and specimens examined] indicate that *Leucochrysa (Leucochrysa) varia* occurs from the western, lowland regions of the Amazonian drainage basin of Brazil, Ecuador and Peru, throughout much of forested Brazil (coastal and inland), and in northeastern, mid-elevation regions of Argentina. The specific areas that we have confirmed include: **Argentina:** Province of Salta. **Brazil:** States of Bahia, Distrito Federal, Mato Grosso, Minas Gerais, Pará, Rio de Janeiro, Rondônia,São Paulo. **Ecuador:** Provinces of Napo, Orellana. **Peru:** District ofMadre de Dios.Confirmed published records include: Freitas and Penny (2001: 280, as *Leucochrysa (Leucochrysa) ampla*), and Mantoanelli et al. (2006: 8).

Unconfirmed, published records from South America include --- Brazil: EspíritoSanto (Navás 1922b: 89), Mato Grosso (Navás 1932: 57); Pará (Navás 1912-1913: 314), Rio de Janeiro (Navás 1922a: 392; Navás 1926: 12; Navás 1929a: 862), Paraguay: San Bernardino [Cordillera] (Navás 1913: 157), and Bolivia: Buenavista [Santa Cruz] (Navás 1928b: 111). The Brazilian records of *Leucochrysa (Leucochrysa) varia* and *Leucochrysa (Leucochrysa) walkerina* by Freitas and Penny (2001: 282) were based on misidentified specimens; thus, only their records for *Leucochrysa (Leucochrysa) ampla* [= *Leucochrysa (Leucochrysa) varia*] are included here.

*Leucochrysa (Leucochrysa) varia* was reported from British Guiana, Suriname, Central America, and Mexico; however we have not confirmed any of these records. Banks (1945: 168) questioned the Navás (1928a: 131) record for Central America (Guatemala). Based on our study here, we also question this record. We confirmed that the Banks (1944: 31) record for British Guiana was in error; a specimen in the AMNH with the data he reported is probably an undescribed species; its abdomen is missing. The specimen(s) that he identified as *Leucochrysa (Leucochrysa) varia* from Suriname (Banks 1944: 31) were not found in the MCZ (P. Perkins, personal communication), nor in the AMNH. This record probably applies to *Leucochrysa (Leucochrysa) pretiosa* or another, undescribed species. In summary, we have not seen specimens of *Leucochrysa (Leucochrysa) varia* from northern South America, the Caribbean region, Central America, or Mexico.

#### Type specimens and rationale for taxonomic changes.

*Chrysopa varia*. Holotype, by original designation, MCZ, male (examined).

**Figure 1.**
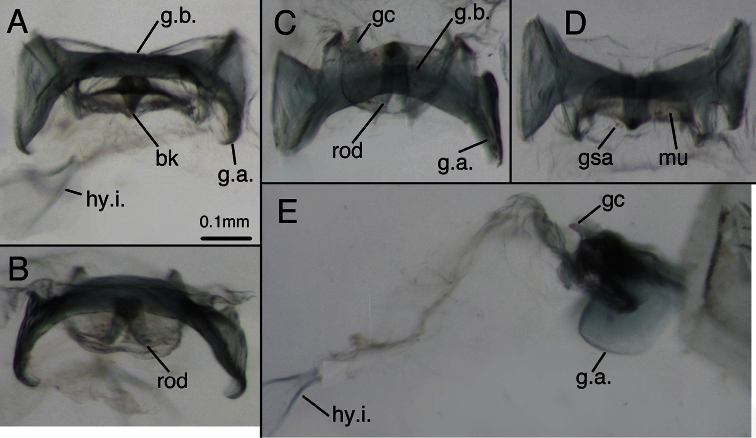
Holotype, *Chrysopa varia* [= *Leucochrysa (Leucochrysa) varia*], male, MCZ, gonarcal complex. **A** Frontal **B** Posterior **C** Ventral **D** Dorsal **E** Lateral. Scale applies to all images. *Abbreviations*: **bk** beak-like tip of mediuncus **gc** gonocornu **gsa** gonosaccus **g.a.** gonarcal apodeme **g.b.** gonarcal bridge **hy.i.** hypandrium internum **mu** mediuncus **rod** mediuncal rod.

The type is in fairly good condition. Its primary label is hand-written, apparently in Schneider’s hand; it reads: “varia nov. sp. / Schneider / Brasilia”. The exact collection site is unknown. In addition to Fig. 1 here, images of the external and male features are in the MCZ Type Database (website: http://insects.oeb.harvard.edu/MCZ/index.htm).

*Chrysopa internata*. Holotype by monotypy, BMNH, male (examined).

Walker originally referenced two specimens under the name *Chrysopa internata* (“*a*” from Brazil and “*b*” without locality data); he also stated that *Chrysopa internata* had two varieties. Because one of the specimens must represent Walker’s lettered variety (“var. β”), that specimen must be excluded from the type series of *Chrysopa internata* under Art. 72.4.1 (see Oswald 2007). Kimmins (1940: 444) identified the specimen without locality data (specimen “*b*”) as the primary *Chrysopa internata* type (a lectotype). Because the actual type series of *Chrysopa internata* consists of only a single specimen, his lectotype designation was unnecessary; we recognize Kimmins’ action as identification of the holotype.

The excluded “var. β” specimen (specimen “*a*” from Brazil), was subsequently designated the type of *Leucochrysa walkerina* Navás (see below).

**Figure 2.**
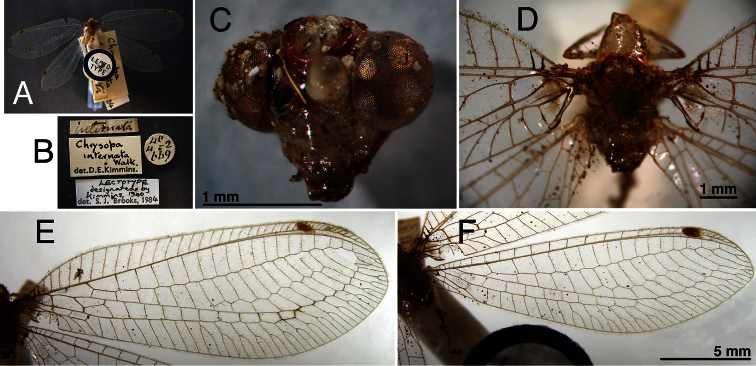
Holotype, *Chrysopa internata* [= *Leucochrysa (Leucochrysa) varia*], male, BMNH, external features. **A** Habitus **B** Labels **C** Head, frontal **D** Thorax, base of wings, dorsal **E, F** Forewing and hindwing; scale applies to both wings.

**Figure 3.**
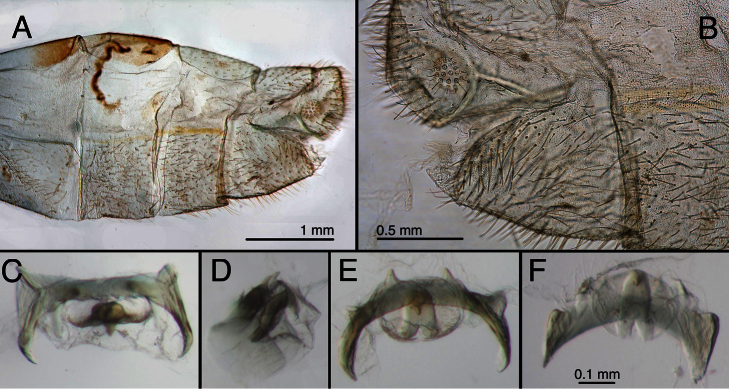
Holotype, *Chrysopa internata* [= *Leucochrysa (Leucochrysa) varia*], male, BMNH, terminalia. **A** Abdominal segments 5–9, lateral **B** Terminal segments, lateral **C** Gonarcal complex, frontal **D** Gonarcal complex, lateral **E** Gonarcal complex, frontoventral **F** Gonarcal complex, ventral; same scale applies to **C–F**.

The *Chrysopa internata* holotype is in fairly good condition; the head is separated from the body and the dissected abdomen is in glycerin, in a vial attached to the pinned specimen. The labels are hand-written and printed (Fig. 2B). The specimen carries no locality data; its collection site is unknown. All of its features, including the male terminalia, correspond to those of the *Leucochrysa (Leucochrysa) varia* type (see Figs 2–3).

*Chrysopa ampla*. Holotype by monotypy, BMNH, female (examined).

Walker originally referenced two specimens (“*a*” and “*b*”) under the name *Chrysopa ampla*; he also stated that *Chrysopa ampla* had two varieties. Because one of the specimens must represent Walker’s lettered variety (“var. β”), that specimen must be excluded from the type series of *Chrysopa ampla* under Art. 72.4.1 (see Oswald 2007). Thus, the actual type series of *Chrysopa ampla* consists of only a single specimen, the one that was listed by Walker as “var. α” and that carries no locality data. Therefore, this specimen constitutes the holotype by monotypy. This designation is consistent, nomenclaturally, with the action by Kimmins (1940: 444), who considered the specimen as the name-bearing type when he designated it as the “lectotype”.

The excluded “var. β” specimen was reported from “Georgia”. Tauber (2004: 1132) identified it as *Leucochrysa (Leucochrysa) insularis* Walker, and it bears her label with that name.

**Figure 4.**
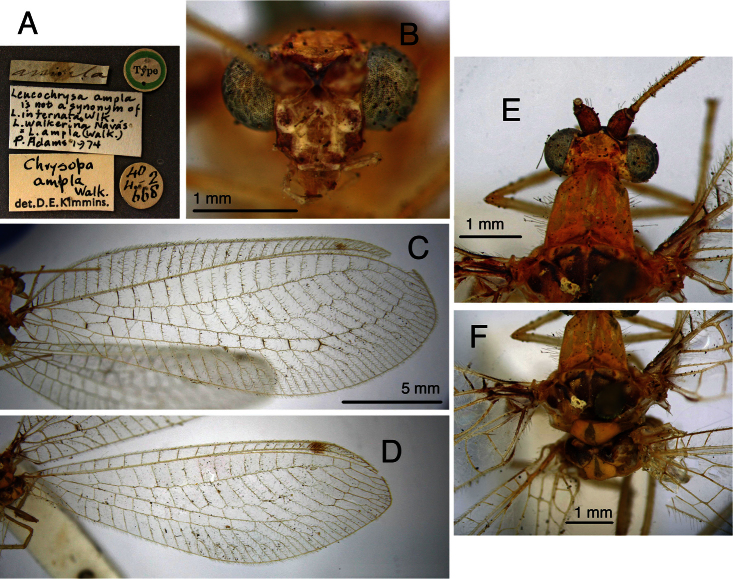
Holotype, *Chrysopa ampla* [= *Leucochrysa (Leucochrysa) varia*], female, BMNH, external features. **A** Labels **B** Head, frontal **C, D** Forewing and hindwing; scale applies to both wings **E** Head and prothorax, dorsal **F** Mesonotum, metanotum, base of wings.

**Figure 5.**
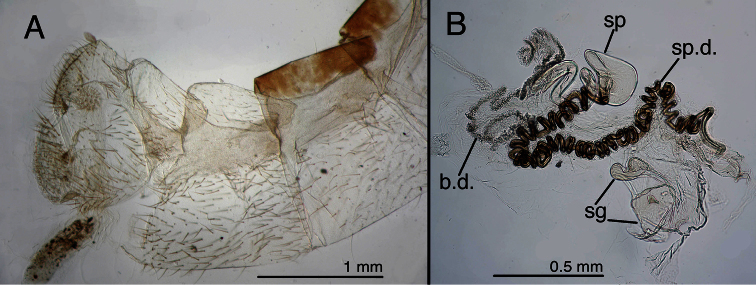
Holotype, *Chrysopa ampla* [= *Leucochrysa (Leucochrysa) varia*], female, BMNH, abdomen (teneral). **A** Abdominal segments 6–9, lateral **B** Genitalia. *Abbreviations*: **b.d.** bursal duct **sg** subgenitale **sp** spermatheca **sp.d.** spermathecal duct.

The *Chrysopa ampla* holotype is in fairly good condition; the dissected abdomen is in glycerin, in a vial attached to the pinned specimen. The labels are hand-written (not by Navás) and printed (Fig. 4A). The specimen carries no locality data; its collection site is unknown. All of the features (external and genitalic) of this type correspond to those of female *Leucochrysa (Leucochrysa) varia* (see Figs 4–5).

*Leucochrysa vegana*. Type(s) by original designation, probably missing.

Navás (1917: 278), in his original description, did not indicate the depository or sex of the type; he reported that it was from the “Coll. Br. [Brother] Apolinar Maria [María]” (not examined). It was not found in the Navás collection (Monserrat 1985: 240), the BMNH, the MNHN, or the MCZ. And despite searches by colleagues (see acknowledgements) in Colombia where Br. Apolinar María lived, it was not found. Apparently, the Br. Apolinar María collection was housed in the Museo de la Universidad de la Salle and was destroyed during a political upheaval in 1948.

Navás mentioned the similarity between *Leucochrysa vegana* and *Leucochrysa varia* (“*variae* Schn.”), but he did not point out why he considered that *Leucochrysa vegana* was different. Most of the features that he described for *Leucochrysa vegana* are found on *Leucochrysa (Leucochrysa) varia* specimens, including the markings at the base of the *Leucochrysa vegana* forewing that he illustrated. Thus, although it is unlikely that Banks actually saw the *Leucochrysa vegana* type, his synonymy (based on Navás’ description) made sense at the time.

The type locality of *Leucochrysa vegana* (La Vega, which is in the Cordillera Oriental of Colombia), is considerably north of the currently known northern limit of *Leucochrysa (Leucochrysa) varia* [the western regions of the Amazon drainage basin of Ecuador and Peru (e.g., the Yasuní Reserve in the Province of Napo, Ecuador and the Tambopata district of Peru)], but well within the ranges of other, undescribed *Leucochrysa (Leucochrysa) varia*-like species. At this time, we suspect that *Leucochrysa vegana* is not synonymous with *Leucochrysa (Leucochrysa) varia*. However, the region remains very poorly collected, so it is possible that our suspicion is in error. Thus, while we await the collection of *Leucochrysa (Leucochrysa) varia* from La Vega or nearby, to confirm Banks’ synonymy, we consider the species name to be a nomen dubium.

*Leucochrysa phaeocephala*. Type(s), by original designation, Hamburg, probably destroyed during W.W. II, sex unknown (not examined).

Banks’ (1944: 30) synonymy was made on the basis of the description; it does not appear that he saw the type. Navás (1929b: 21) reported that the specimen was collected in Dutch Guiana in 1908 (now, Suriname). Because we have found no records of *Leucochrysa (Leucochrysa) varia* from the northern regions of South America, we reverse Bank’s synonymy; we will deal with the species in a later publication.

*Leucochrysa walkerina*. Holotype by monotypy, BMNH, female (examined).

Navás (1913: 102) considered that Walker’s *internata* variety β represented a distinct species; he explicitly applied the species name “*walkerina*” in honor of Walker. Kimmins (1940: 444) designated Walker’s specimen “*a*” of *internata* (labeled from Brazil) as the type of *Chrysopa internata* variety β; thus it became the holotype of *Leucochrysa walkerina* (see Oswald 2007).

**Figure 6.**
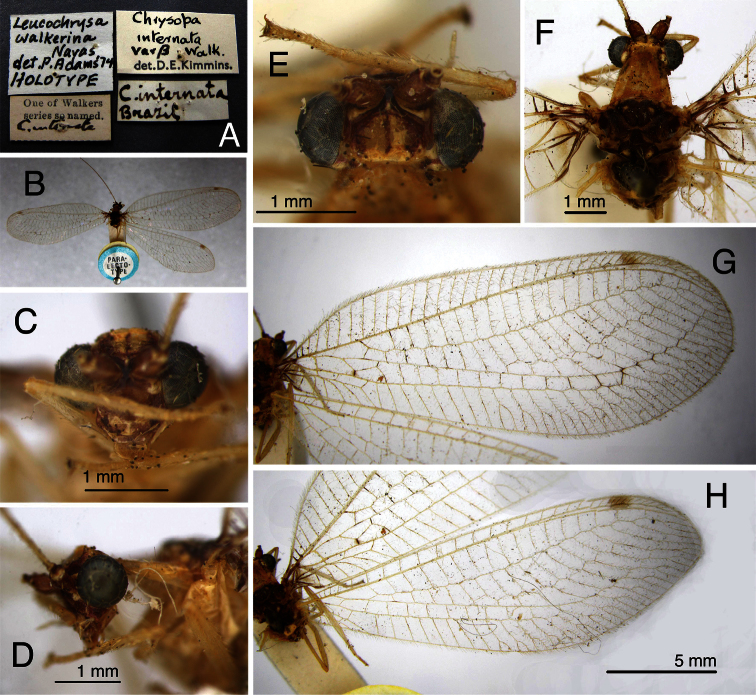
Holotype, *Leucochrysa walkerina* [= *Leucochrysa (Leucochrysa) varia*], female, BMNH, external features. **A** Labels **B** Habitus **C** Head, frontal **D** Head, lateral **E** Head, dorsal **F** Head, thorax, dorsal **G & H** Forewing and hindwing; scale applies to both wings.

**Figure 7.**
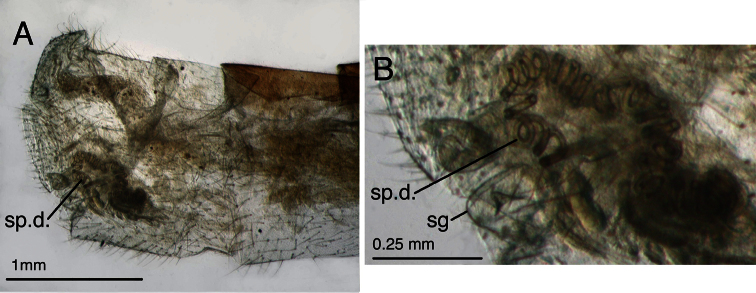
Holotype, *Leucochrysa walkerina* [= *Leucochrysa (Leucochrysa) varia*], female, BMNH, abdomen and genitalia. **A** Terminal segments, genitalia visible within abdominal cavity **B** Enlargement of genital structures [Note the spermathecal duct and subgenitale.]. *Abbreviations*: **sg** subgenitale **sp.d.** spermathecal duct.

The *walkerina* type is slightly teneral, but in good condition; the cleared abdomen is in glycerine, in a vial attached to the specimen. The pin carries four labels below the specimen (including a locality label, “Brazil”) (Fig. 6A); it also has a “Paralectotype” label above the specimen (Fig. 6B). The external characteristics (Fig. 6) are consistent with those of *Leucochrysa (Leucochrysa) varia*, and the elongate, coiled, spermathecal duct is that of *Leucochrysa (Leucochrysa) varia* (Fig. 7).

#### Diagnosis.

**Figure 8.**
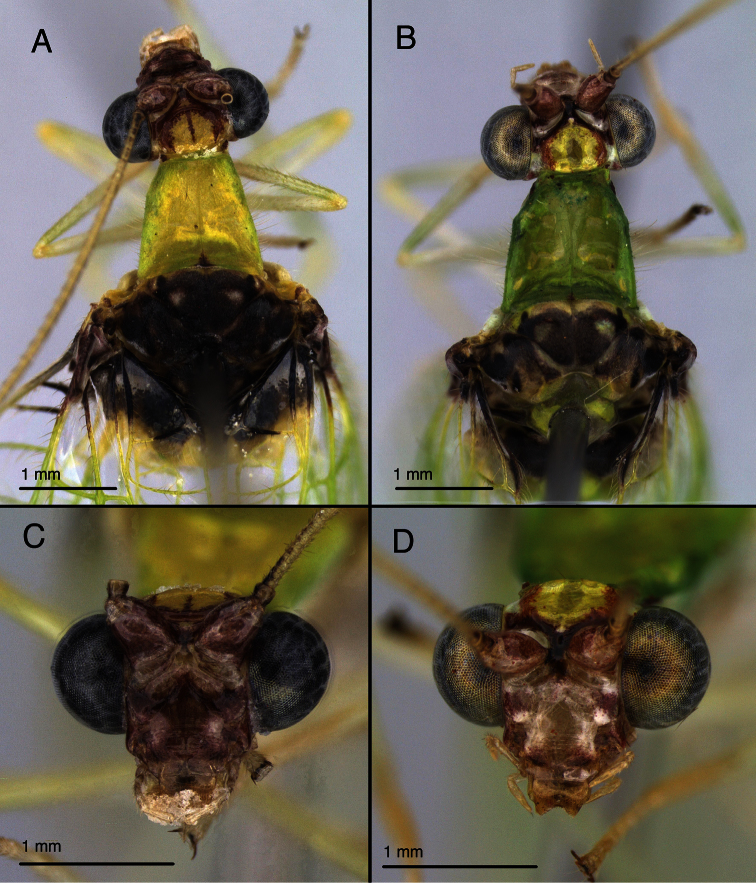
Variation in *Leucochrysa (Leucochrysa) varia* head and thoracic markings. **A, B** Head, thorax, dorsal **C–D** head, frontal (all, state of Minas Gerais, Brazil).

As the name *varia* implies, adults of *Leucochrysa (Leucochrysa) varia* exhibit a wide range of color variation; there are black and red morphs, with and without coloration on the mesoscutellum (See Fig. 8 here, Fig. 7 in Mantoanelli et al. 2006). In addition, preserved specimens of *varia*-like species tend to loose their natural coloration very quickly; old specimens are especially discolored. Thus, it is important to emphasize that accurate identification of the *varia*-like species can only be made by careful examination of the male or female genital characters.

Externally, *Leucochrysa (Leucochrysa) varia* and *Leucochrysa (Leucochrysa) pretiosa* adults usually (but not always) can be separated by differences in the darkness of certain veins and the degree of suffusion around the veins. Both species have black to dark brown suffusion surrounding the second m-cu crossvein, but the marking generally is less prominent in *Leucochrysa (Leucochrysa) varia* than in *Leucochrysa (Leucochrysa) pretiosa*. Moreover, in both species the distal two to four Psm–Psc crossveins and at least the three basal outer gradates are darkened and the membranes surrounding these veins are shaded to some degree. In *Leucochrysa (Leucochrysa) varia*, the degree of darkening and suffusion around these veins is generally uniform, whereas in *Leucochrysa (Leucochrysa) pretiosa*, the distal Psm-Psc crossvein is usually darker and more prominent than the other Psm-Psc crossveins or the outer gradates.

In *Leucochrysa (Leucochrysa) varia* males, the sclerotized mediuncal plate is elongate; its rods are narrow and parallel; its membranous connection to the gonarcal bridge does not extend laterally beyond the gonocornua and is soft [not broad, leathery and stiff, as in *Leucochrysa (Leucochrysa) pretiosa*]. The *Leucochrysa (Leucochrysa) varia* female is distinguished by a very long, strongly coiled spermathecal duct and a spermatheca that is scoop-shaped distally and has a convoluted, tubular, basal section leading to a broad, fluted bursal duct.

#### Redescription.

*Head* (Fig. 8): 1.8–1.9 mm wide (including eyes). Frons, clypeus cream to red, with anterior clypeal margin dark red; gena red to reddish brown; maxillary, labial palp yellowish to cream-colored. Vertex with central area raised, yellowish to cream-colored, with prominent, dark red-wine-colored, V-shaped mark along anterior margin; lateral margin, midline sometimes also with red-wine-colored marks. Antenna: dorsum of scape lightly to darkly tinged with red-wine color; pedicel dark red to ivory-colored, with inner margin darkened; flagellum cream-colored, with amber bristles; inner margin of basal ca 3 flagellomeres tinged with red to black; antennal fossa marked with red laterally.

*Thorax* (Fig. 8): Cervix lightly tinged with red laterally. Pronotum 1.2–1.5 mm long, 1.2–1.5 mm wide, yellowish to greenish, unmarked except for small posteromesal red to black mark. Mesonotum, metanotum mostly dark, variable, with four types of color morphs (black entire, black open, red entire, red open) [see Fig. 8 and Mantoanelli et al. 2006].

*Wings* (Fig. 9A–B): Forewing 17.3–20.5 mm long, 6.5–7.8 mm wide (at widest point); ratio of length: maximum width = 2.6–2.7:1. Costal area moderately broad; tallest costal cell (#9–10) 1.5–1.9 mm tall, 2.6–3.3 times width, 0.3 times width of wing (midwing). First intramedian (im1) cell quadrangular, width (anterior margin) 1.4–1.6 times width (anterior margin) of third median cell (m3), 2.3–3.2 times length of posterior margin of m3, length of basal vein (= ma, median arculus) 1.0–1.1 times greater than length of distal vein. First radial crossvein distal to origin of radial sector (Rs); radial area (between R and Rs) with single row of 16–18 closed cells; tallest cell (#6–8) 2.16–2.58 times taller than wide. No crassate veins; 5–7 b cells (= cells beneath Rs, not including an inner gradate vein). Two series of gradate veins; 6–11 inner gradates, 7–9 outer gradates. Height of fourth gradate cell 3.6–5.4 times width. Eight to nine b’ cells (cells beneath Pseudomedia after im2). Three intracubital cells (two closed). Membrane mostly clear except basal area with small, reddish brown patch, stigma opaque to light brown, with large very dark brown mark basally, small, light brown clouding around second m-cu crossvein and around distal Psm–Psc crossveins, sometimes with clouding around distal leg of im1 and around crossveins between distal b’ cells. Veins mostly green, except anterior tips of most costal crossveins, base of Radius, bases of ca three to five radial crossveins, distal three to four Psm–Psc crossveins, outer gradates, and forks of posterior marginal veins darkened to black; inner gradates, posterior veins of distal three to four b’ cells slightly darkened. Hindwing 16.2–18.3 mm long, 5.3–6.0 mm wide. Two series of gradate veins; 6–10 inner, 6–8 outer; 14–17 radial cells (counted from origin of R, not false origin). Four to six b cells (including small b1 cell); six to eight b’ cells beyond im2; two intracubital cells (one closed). Membrane clear; stigma with pronounced dark brown mark basally. Veins mostly light green; middle costal crossveins, outer gradates, bases of marginal forks, black.

**Figure 9A.**
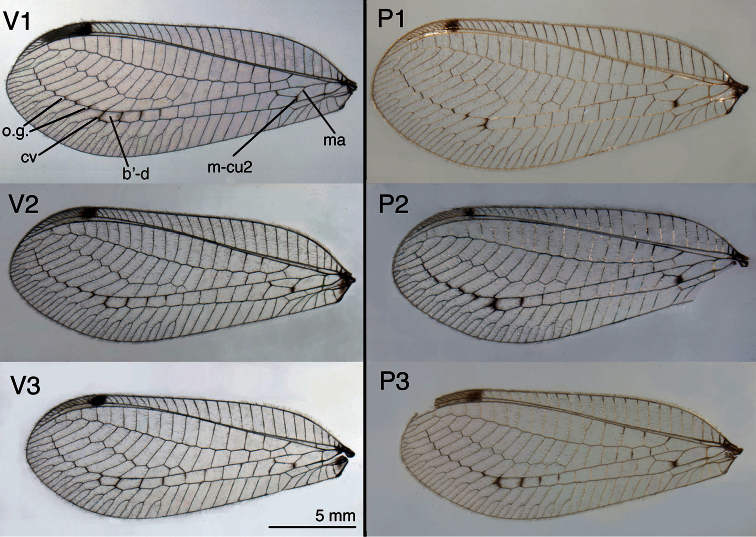
Forewings from three specimens each of *Leucochrysa (Leucochrysa) varia* and *Leucochrysa (Leucochrysa) pretiosa*, illustrating interspecific and intraspecific variation in markings, size, and shape **Left** *Leucochrysa (Leucochrysa) varia* (V1. Salta, Argentina; V2. Rio de Janeiro, Brazil; V3 Napo, Ecuador) **Right** *Leucochrysa (Leucochrysa) pretiosa* (P1. Capital District, Venezuela; P2. Carabobo, Venezuela; P3. Trinidad Island, Trinidad & Tobago). *Abbreviations*: **b’-d** distal cell in lower Banksian series **cv** distal Psm-Psc crossvein **ma** median arculus (= basal vein of im1) **m-cu2** distal (= second) m-cu crossvein **o.g.** basal three outer gradates.

**Figure 9B.**
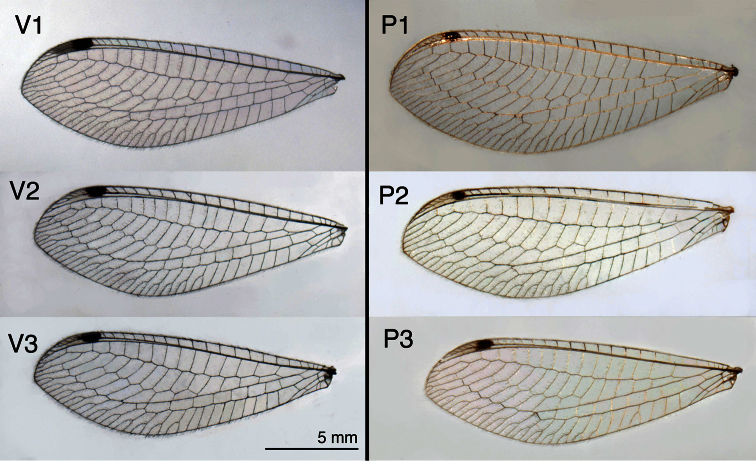
Hindwings from three specimens each of *Leucochrysa (Leucochrysa) varia* and *Leucochrysa (Leucochrysa) pretiosa*. Same information as Fig. 9A.

**Figure 10.**
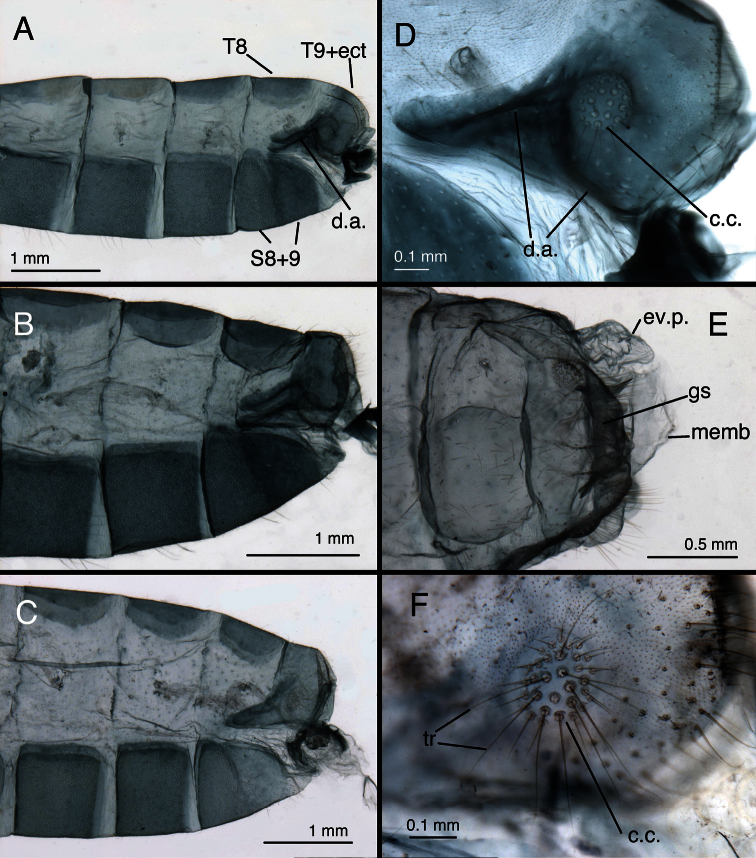
*Leucochrysa (Leucochrysa) varia* male abdominal structures. **A, B, C** Terminal segments, lateral, demonstrating intraspecific variation in the shape, expansion and sclerotization of the abdomen **D** Tergite 9+ectoproct **E** Abdominal segments 8 & 9, dorsal **F** Callus cerci. (A–D State of Rio de Janeiro, Brazil; E–F Province of Orellana, Ecuador). *Abbreviations*: **c.c.** callus cerci **d.a.** dorsal apodeme of ninth tergite+ectoproct **ev.p.** eversible membranous pouch **gs** gonarcus **memb** membrane **S8+9** fused eighth and ninth sternites **tr** trichobothria **T8** eighth tergite **T9+ect** fused ninth tergite and ectoproct.

**Figure 11.**
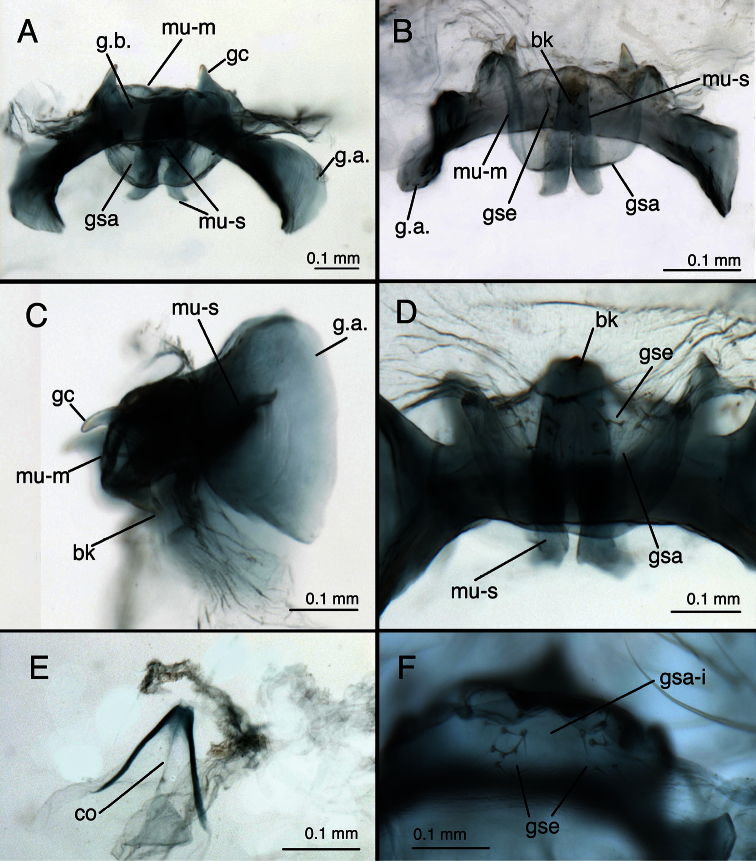
*Leucochrysa (Leucochrysa) varia* male genitalia. **A** Gonarcal complex, dorsal **B** Gonarcus, ventral **C** Gonarcus, lateral **D** Mediuncus, ventral **E** Hypandrium internum **F** Gonosetae inside gonosaccus. (A, C–F State of Rio de Janeiro, Brazil; B Province of Orellana, Ecuador). *Abbreviations*: **bk** beak-like tip of mediuncus **co** comes **gc** gonocornu **gsa** gonosaccus **gsa-i** interior of gonosaccus **gse** gonosetae **g.a.** gonarcal apodeme **g.b.** gonarcal bridge **mu-m** mediuncal membrane **mu-s** sclerotized section of mediuncus (mediuncal rod).

**Figure 12.**
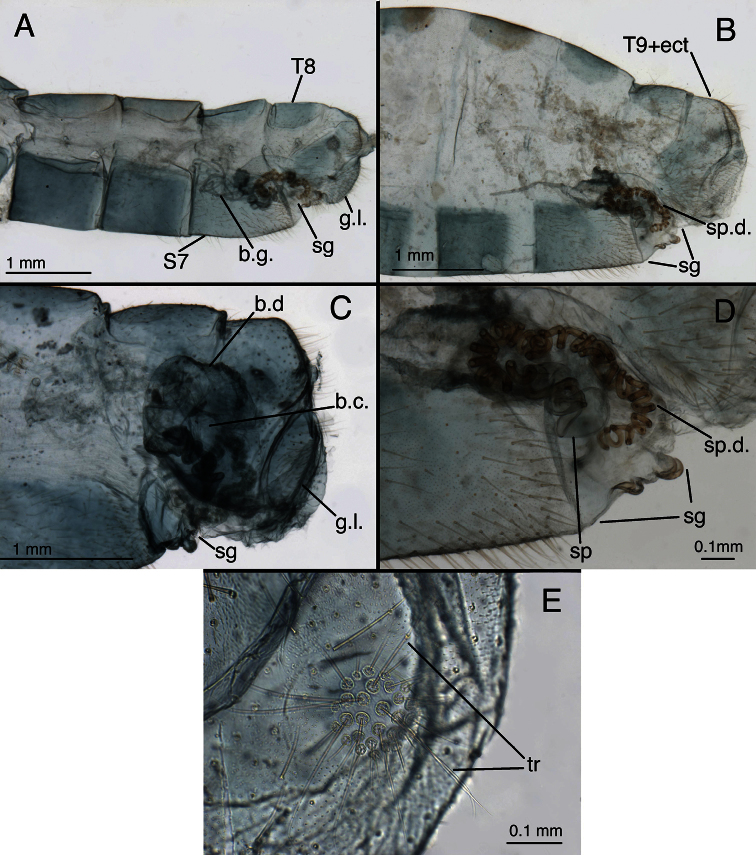
*Leucochrysa (Leucochrysa) varia* female abdominal structures. **A, B, C** Terminal segments, lateral, demonstrating intraspecific variation in the shape and expansion of the abdomen **D** Genitalia, lateral **E** Callus cerci and trichobothria. (A–B, D District of Madre de Dios, Peru; C, E State of Rio de Janeiro, Brazil). *Abbreviations*: **b.c.** bursa copulatrix **b.d.** bursal duct **b.g.** bursal gland **g.l.** gonapophysis lateralis **sg** subgenitale **sp** spermatheca **sp.d.** spermathecal duct **S7** seventh sternite **tr** trichobothria **T8** eighth tergite **T9+ect** fused ninth tergite and ectoproct.

**Figure 13.**
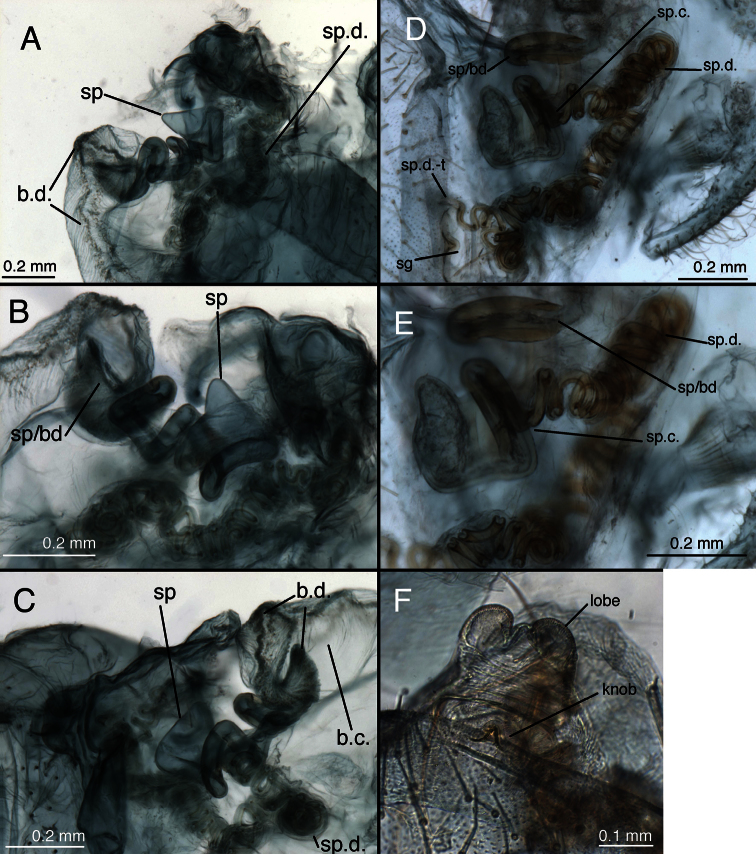
*Leucochrysa (Leucochrysa) varia* female genitalia. **A–E** Spermatheca complex with a variety of views and magnifications **F** Subgenitale (all, State of Rio de Janeiro, Brazil). *Abbreviations*: **b.c.** bursa copulatrix **b.d.** bursal duct **knob** protruding knob on surface of subgenitale **lobe** distal lobe of subgenitale **sg** subgenitale **sp** spermatheca **sp/bd** connection between spermatheca and bursal duct **sp.c.** spermathecal connection to spermathecal duct **sp.d.** spermathecal duct **sp.d.-t** brushy terminus of spermathecal duct in subgenitale.

**Figure 14.**
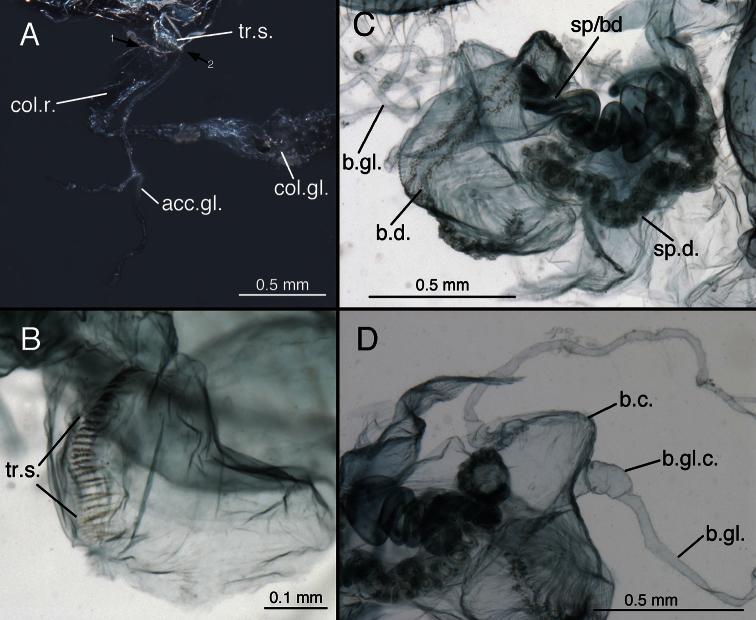
*Leucochrysa (Leucochrysa) varia* female genitalia. **A** Colleterial and accessory glands [Arrow 1 indicates the connection of the duct from the colleterial reservoir to the shelf of the transverse sclerite. Arrow 2 indicates the connection of the duct from the accessory gland to the shelf of the transverse sclerite.] **B** Transverse sclerite **C** Genitalia, including bursal glands **D** Tip of bursa copulatrix with pipe-fitting-like connection to bursal gland(all, State of Rio de Janeiro, Brazil). *Abbreviations*: **acc.gl.** bifurcated accessory gland **b.c.** bursa copulatrix (tip) **b.d.** bursal duct **b.gl.** bursal gland **b.gl.c.** connection between bursal gland and bursa copulatrix **col.gl.** colleterial gland **col.r.** colleterial reservoir **sp/bd** connection between spermatheca and bursal duct **sp.d.** spermathecal duct **tr.s.** transverse sclerite.

*Abdomen* (Figs 1, 10–14): Sternites, tergites with long, slender setae throughout, microsetae moderately dense; pleural region with setae small, very sparse, microsetae very small. Rim around each sternite heavily sclerotized, especially anteriorly, fading posteriorly. Tergites narrow, roughly rectangular, with rounded margins. Spiracles oval externally; atria not enlarged. Sternites S2–3 longer than tall; S5–7 more square-shaped; distal segments (beyond A4) expanded, height of pleural region greater than height of sternites. Coloration: mostly green, with yellow mesally. Tergites T5, T6 with large black spots, bordered by red; callus cerci white; setae, trichobothria golden.

*Male*: S6 height and length approximately equal, S7 height ca 1.1–1.2 times length (lateral view); S4–S8 with dense microtholi, S3 with microtholi laterally, sometimes also across entire posterior region, absent or sparse anteriorly, mesally; S1–2, S9 without microtholi. Callus cerci round to slightly oval (ca 1.1–1.2× taller than wide), diameter ca 0.16–0.28 mm, with ca 30–35 trichobothria of variable length. T9+ectoproct rounded posterodorsally, truncate to rounded distally, broadly fused mesally, midline with small distal cleft, with long setae; ventral section tapering, rounded proximally, extending above S8+9 only to suture between S8 and S9; dorsal apodeme substantial, but not thick, straight basally, forking midway on anterior margin of callus cerci; dorsal branch extending around dorsal margin of callus cerci to midway on posterior margin, ventral branch curving ventrally well below callus cerci, then bending posteriorly, extending along ventral margin of ectoproct. S8+9 fused, with trace of suture dorsally, with clear intersegmental demarcation throughout; S8 tall (1.5–2.0× taller than long), ca one-half (0.44–0.47×) length of S8+9 along ventral margin; S8+9 (lateral view) with proximal margin slightly convex, dorsal surface of S8 rounded, of S9 curved distally, steeply sloped at terminus; terminus without knob or gonocristae; membranous region above terminus of S9 with pair of large, eversible, lateral pouches. Gonarcus well sclerotized, widely arcuate (total span, 0.67–0.84 mm); bridge broad (0.38–0.62 mm long), dorsoventrally flattened, gently curved throughout; lateral apodeme rounded dorsally, more acute ventrally (0.21–0.24 mm wide; 0.29–0.33 mm tall); gonocornua extending forward from anterior edge of gonarcal bridge, basally stout, with ventral, straight projection, tapering to narrow, rounded apex (length, 0.09–0.15 mm); distance between inner bases of gonocornua 0.15–0.25 mm, distance between tips 0.20–0.26 mm. Mediuncus located beneath, and well separated from gonarcus, with mesal, recurved beak, lateral, well-sclerotized, stiff membranous, curved arms; mediuncus attached to gonarcal bridge via robust membrane that extends along width of gonarcal bridge between outside margins of gonocornua, folds beneath gonarcal bridge and beneath mediuncal beak to form deep gonosaccus; gonosaccus with two fields of five to seven short gonosetae on chalazae. Hypandrium internum: arm 0.25–0.37 mm long, distal span between arms 0.24–0.32 mm.

*Female*. Height of S6 ca 0.75 times length, S7 height ca 0.60 times length. Callus cerci round, diameter 0.17–18 mm, with 30–35 trichobothria. T8 roughly quadrate (lateral view) with rounded corners, similar in depth to T6. T9+ectoproct elongate, slanting anteriorly; ventral margin slightly convex, extending slightly below level of gonapophyses laterales. Dorsal margin of S7 with slight taper basally, becoming more pronounced distally; terminus unmodified, with terminal (posteroventral) setae slightly more dense than in other areas. Gonapophysis lateralis rounded to slightly acute dorsally, rounded distally, ventrally, ca 0.53–0.60 height of T9+ectoproct; inner membranous surface not expandable, with ca two vertical rows of short setae. Colleterial complex consisting of membranous gland connected to colleterial reservoir via broad duct, and elongate ribbon-like accessory gland; colleterial gland elongate, delicate, transparent; colleterial reservoir smaller, delicate, transparent, tapering to narrow, granulose, spiny duct; accessory gland narrow, elongate, forked distally, with spiny surface; accessory gland and colleterial duct connected to lightly sclerotized, widened, flattened platform extending from below transverse sclerite; transverse sclerite curved, lightly sclerotized, slender throughout, with long teeth (setae?). Spermatheca with initial (posterior) section scoop-shaped, broad, thick, tapering slightly at base (ca 0.25 mm wide along distal margin x ca 0.20 mm height from tip of distal margin to base of scoop), with elongate, broad, smooth, convoluted tube extending down one side, looping in U-shaped turn, then twisting through several loops before joining bursal duct [tube ca 1.1 mm in total length, ca 0.05 mm in width throughout]; spermathecal invagination not specifically identified. Spermathecal duct extremely long, well sclerotized throughout, densely, tightly coiled, arising from side of scoop-shaped section of spermatheca; coiled length ca 3–5 mm, including membranous, brushy, less coiled distal section, uncoiled length much greater. Bursal duct extending from tip of tubular spermathecal velum, basal section membranous, broad, curved, fluted; surface with striated folds, lateral margins of major folds heavily granulose. Bursa copulatrix small, saccular, extended over spermatheca, slightly into section of S7; ventral surface with small striated folds; dorsal surface smooth; pair of clear, elongate, tubular bursal glands attached dorsally to base of bursa via clear, pipe-fitting-like bases; bursal glands very long, unbranched; surface lightly granulose. Subgenitale with smooth (unfolded), rounded surface, broad, rectangular, robust, bilobed projection extending distally at ca 90° angle to subgenitale surface, lobes large, with minute setae on surface, region between lobes extending distally as smaller, acute lobe; base of bilobed projection with dense transverse folding, with sclerotized, knob-like mesal lobe projecting from pair of scalloped, sclerotized arms.

#### Larva.

All instars described (Mantoanelli et al. 2006).

#### Biology.

Developmental and survival rates of immature stages under five constant temperatures and larval trash-carrying behavior studied by Mantoanelli et al. (2006) and Mantoanelli and Albuquerque (2007).

#### Variation.

The coloration of the head, mesonotum and metanotum is the most obvious variation expressed by *Leucochrysa (Leucochrysa) varia* [see above]. However, color is not the only feature that varies among *Leucochrysa (Leucochrysa) varia* specimens. For example, the expansion of abdominal segments 4–9 and the degree of sclerotization of the integument varies greatly among both male and female specimens (see Figs 10, 12). Differences in sclerotization are particularly noticeable in the ventral apodemes of the male T9+ectoproct (Fig. 10). We suspect that these features (abdominal expansion and integumental sclerotization) are at least partially a function of age and developmental or reproductive maturation.

Two features, specific to males, also show considerable variation. First, the breadth of the elongate mediuncal “rods” varies. For example, the *Chrysopa internata* type [= *Leucochrysa (Leucochrysa) varia*] has very broad mediuncal rods (Fig. 3), whereas the *Chrysopa varia* type [= *Leucochrysa (Leucochrysa) varia*] has considerably narrower ones (Fig. 1). Other specimens express a range in size between these two specimens (Fig. 11). Finally, the pattern of microtholi on the third abdominal sternite varies among males. In one specimen from Ecuador, the microtholi were restricted to a relatively small lateral patch; they were absent from the mesal region of the sternite. In another specimen, this one from Brazil, microtholi were present along the entire lateral region of the sternite and also in a strip along the posterior margin. Whether this variation has a geographical component is unknown.

#### Material examined

**(in addition to the types listed above). Argentina. *Salta***: Cafayate, ii–iv.1968, Hayward (2♂, 3♀, USNM). **Brazil**. ***Mato Grosso***: Itiquira [17°12'S, 54°09'W], 522 m, 17.i.1996, 2♂, V. Cruz; idem, 26.viii.1996, 2♂, V. Cruz; idem, 5.x.1996, 1♂, V. Cruz; 18.x.1996, 1♂, 3♀, V. Cruz; idem, 31.xii.1996, 2♂, V. Cruz; idem, 13.i.1997, 1♀, S. Freitas (all FCAV-UNESP). ***Minas Gerais***: Caratinga, 19.ix.2011, S. Freitas & F. Sosa (2♂, 2♀, UCOB). ***Pará***: Rio Xingu Camp, ca 60 km S. Altamira [3°39'S, 52°22'W], 2–8.x.1986, malaise trap, night collection, P. Spangler & O. Flint (1♀, USNM). ***Bahia***: Camacan, Reserva Serra Bonita, Fazenda Paris, 200 m, 6.x.2005, G. S. Albuquerque, M. J. Tauber, C. A. Tauber Expedition, Oct. 2005 (15♂, 11♀, UCB). ***Distrito Federal***: CENARGEN Farm nr. Núcleo Bandeirante, 20.iii.1999, M. J. & C. A. Tauber (2♂, 3♀, UCB); idem, 9.v.2002 (2♂, UCB). ***Rio de Janeiro***: Parque Estadual do Desengano, Santa Maria Madalena, Terras Frias, 30.iii.1999, M. J., C. A. & P. J. Tauber, G. S. Albuquerque (3♂, 2♀, UCB); idem, 15.v.2002, M. J., C. A., P. J. & A. J. Tauber, G. S. Albuquerque, E. A. Silva (9♂, 6♀, UCB); idem, 28.x.2003, G. S. Albuquerque, M. J. Tauber, C. A. Tauber Expedition, Oct-Nov 2003 (1♀, UCB); idem, 22.iv.2004, G. S. Albuquerque, M. J. Tauber, C. A. Tauber Expedition, April 2004 (16♂, 10♀, UCB); Parque Estadual do Desengano, Campos dos Goytacazes, Babilônia, 26.iii.1999, M. J., C. A. & P. J. Tauber, G. S. Albuquerque (9♂, 6♀, UCB); idem, 21 & 27.iii.2001, M. J., C. A. & P. J. Tauber, G. S. Albuquerque (5♂, 3♀, UCB); idem, 20.v.2002, M. J., C. A., P. J. & A. J. Tauber, G. S. Albuquerque, E. A. Silva (6♂, UCB); Parque Estadual do Desengano, Campos dos Goytacazes, Santo Antônio do Imbé, 24 & 31.iii.1999, M. J., C. A. & P. J. Tauber, G. S. Albuquerque (8♂, 2♀, UCB); idem, 27.iii.2001, M. J., C. A. & P. J. Tauber, G. S. Albuquerque (1♂, 1♀, UCB); idem, 1 & 5.v.2003, G. S. Albuquerque, M. J. Tauber, C. A. Tauber Expedition, April-May 2003 (2♂, UCB); Parque Estadual do Desengano, Campos dos Goytacazes, Sossego, 25.iii.1999, M. J., C. A. & P. J. Tauber, G. S. Albuquerque (1♂, 1♀, UCB); Parque Estadual do Desengano, Campos dos Goytacazes, Fazenda Pedrinho, 3.v.2003, G. S. Albuquerque, M. J. Tauber, C. A. Tauber Expedition, Apr.-May 2003 (1♂, UCB); near Parque Estadual do Desengano, Campos dos Goytacazes, Fazenda Boa Vista, M. J., C. A. & P. J. Tauber, G. S. Albuquerque (8♂, 6♀, 1 teneral, UCB); Campos dos Goytacazes, Dist. de Morangaba, Fazenda São Julião, 18.x.2005, M. J. & C. A. Tauber, G. S. Albuquerque (2♂, 1♀, UCB); Campos dos Goytacazes, Conceição de Macabu, Santo Agostinho, 21.v.2002, M. J., C. A., P. J. & A. J. Tauber, G. S. Albuquerque, E. A. Silva (1♂, UCB); Campos dos Goytacazes, Conceição de Macabu, Fazenda Carrapeta, 29.iv–6.v.2003, G. S. Albuquerque, M. J. Tauber, C. A. Tauber Expedition, Apr.–May 2003 (1♂, UCB); idem, 23.iv.2004, G. S. Albuquerque, M. J. Tauber, C. A. Tauber Expedition, April 2004 (11♂, 4♀, UCB); Ilha Grande, 01.vi.2002, G. S. Albuquerque (1♂, UCB). ***São Paulo***: São Vicente [23°57'S, 46°23'W], 916 m, 21.x.2009, S. Freitas (1♀, FCAV-UNESP). ***Rondônia***: 62 km s. Ariquemes, 8–20.xi.1994, W. J. Hanson (1♀, USU); idem, 22–31.x.1997 (1♀, USU). Ecuador. *Orellana* [Napo*]: Yasuní Res. Sta. [0°38'S, 6°36'W [Sic!, probably 76°36’], 250 m, 19–30.x.1996, W. J. Hanson (1♀, USU); idem, 18–30.x.1998, W. J. Hanson (1♀, USU); Misahualli nr. Tena, 3–8.x.1999, S. R. Keller (1♂, USU); idem, 26.viii-2.ix.2000, S. & P. Keller (1♀, USU); idem, 6–19.x.2001, C. Branimer (1♀, USU); Yasuni Biol. Res. Sta. [ca 0°40'S, 76°24'W] 1–7.xi.2002, E. M. Fisher (1♂, 1♀, CAS); Coca [= Puerto Francisco de Orellana] [0°03'S, 79°40'W], v.1965, L. C. Peña (1♂, CAS). **Peru. *Madre de Dios*:** Tambopata, 15 km NE Pto. Maldonado, 200 m, 25.vi.1989, R. A. Leschen #177, ex malaise trap (1♀, SEM); Río Tambopata Res., 30 km (air) sw Pto. Maldonado [12°50'S, 69°20'W], 290 m, 08.xi.1984, T. L. Erwin, canopy fogging 04/01 (1♀ alcohol, USNM); idem, 02.v.1984, T. L. Erwin et al., canopy fogging project 02/03 (1♀ alcohol, USNM); Pakitza, Zone 02 [11°55'48"S, 71°35'18"W], 9.ix.1988, insecticidal fog, canopy/pouteria, BIOLAT 02180413, T. L. Erwin (1♀ alcohol, USNM).

[Note: * The Yasuní Research Station is located in Orellana (a province that was separated from Napo province in 1998). Ecuador’s entire Yasuní Biosphere Reserve (established by UNESCO in 1989) encompasses a large area between the Napo River in the north and west and the Curaray River in the south and east; in terms of biological diversity, it is an extremely rich area.]

### Leucochrysa (Leucochrysa) pretiosa

(Banks, 1910)

http://species-id.net/wiki/Leucochrysa_pretiosa

Figs 9A B 15 26

Allochrysa pretiosa Banks [1910] (1910: 150) original description: [Colombia] “Inmba, Cauca, 1,000 meters, January (Fassl)”. Navás (1913: 157) comparison with *Allochrysa colombia* Banks and *Allochrysa varia*.
Leucochrysa pretiosa (Banks). Navás (1917: 279) first reference to combination; Banks (1944: 31) collection records; Banks (1945: 167-168) comparison with *Leucochrysa varia* and *Leucochrysa vulnerata* (Navás), synonymy of *Leucochrysa variata* (Navás), *Leucochrysa delicata* Navás, *Leucochrysa angrandi* (Navás) with *Leucochrysa pretiosa*, collection records; Banks (1948: 169) collection record; Penny (1977: 23) species list; Adams (1979: 97) species list, record from Mexico considered doubtful.
Leucochrysa (Leucochrysa) pretiosa (Banks). Brooks and Barnard (1990: 276) subgeneric determination, species list; Freitas and Penny (2001: 281, 353, fig. 42) brief redescription, collection records, figures -- based on misidentified specimens in the FCAV-UNESP, not *Leucochrysa (Leucochrysa) pretiosa*; Penny (2002: 194, figs 60-62) brief diagnosis, collection records in Costa Rica, drawings of head, wings; Valencia Luna et al. (2006: 48) collection records, image -- species identification unconfirmed; Oswald (2007) catalog listing.
Allochrysa angrandi Navás [1911] (1911: 278) original description: “Guatemala. Angrand leg.”. Navás (1917: 279, *angrandi* not specifically mentioned) genus synonymized with *Leucochrysa*; Penny (1977: 23, as *Allochrysa angrandi* and *Allochrysa angradi*) listing as synonyms of *Leucochrysa pretiosa*; Legrand et al. (2008: 113) lectotype designation, taxonomic notes.
Leucochrysa angrandi (Navás). Banks (1945: 167) first reference to combination, synonymy with *Leucochrysa pretiosa*; Penny (1977: 23, as *Leucochrysa angradi*) listing as a synonym of *Leucochrysa pretiosa*; Adams (1979: 97) listing as a valid species, without comment, probable occurrence in Mexico; Brooks and Barnard (1990: 276) listing as a synonym of *Leucochrysa (Leucochrysa) pretiosa*, without comment; Oswald (2007) catalog listing, as a synonym of *Leucochrysa (Leucochrysa) pretiosa*.
Leucochrysa (Leucochrysa) angrandi (Navás). **Valid status reinstated.** See below.
Allochrysa variata Navás [1913] (1912-1913: 315) original description: “Panamá: V. de Chiriqui, 25-400 ft., Champion; Mexique: Cuesta de Misantla, M. Trujillo”. Penny (1977: 23, as *Allochrysa varieta*) listing as a synonym of *Leucochrysa pretiosa*.
Leucochrysa variata (Navás). Navás (1917: 279) first reference to combination; Banks (1945: 167) synonymy with *Leucochrysa pretiosa*; Penny (1977: 23, as *Leucochrysa varieta*) listing as a synonym of *Leucochrysa pretiosa*; Brooks and Barnard (1990: 276) listing as a synonym of *Leucochrysa (Leucochrysa) pretiosa*.
Leucochrysa (Leucochrysa) variata (Navás). **Valid status reinstated.** See below.
Leucochrysa delicata Navás [1925] (1925: 190, fig. 18) original description: “Costa Rica: Reventazón, 15 de Marzo de 1923. Janson et Sons. Col. m.”. Banks (1945: 167) synonymy with *Leucochrysa pretiosa*; Adams (1979: 97) species list, note regarding absence of type; Brooks and Barnard (1990; 276) listing as a synonym of *Leucochrysa (Leucochrysa) pretiosa*; Oswald (2007) catalog listing of *Leucochrysa delicata* and *Leucochrysa (Leucochrysa) delicata* as synonyms of *Leucochrysa (Leucochrysa) pretiosa*. Neotype designation below.
Leucochrysa erminea Banks [1946] (1945: 169) original description: “Barro Colorado, Canal Zone, August (F. H. Hull) Type M.C.Z. no. 25657”. Penny (1977: 22) species list; Adams (1979: 97) species list.
Leucochrysa (Leucochrysa) erminea Banks. Brooks and Barnard (1990: 276) subgeneric determination, species list; Oswald (2007) catalog listing. **Syn. n.**

#### Known geographical distribution.

Southern Mexico,northern to southern Central America, the Caribbean region, and northern region of South America (Venezuela, central Ecuador, and western Colombia) [based on confirmed published records and specimens examined]. **Mexico:** Chiapas. **Belize:** Cayo District. **Costa Rica:** Provinces of Cartago, Puntarenas. **Panama:** Canal Zone. **Trinidad & Tobago:** Trinidad Island. **Venezuela:** States of Amazonas, Aragua, Carabobo, Falcón, Lara, Mérida, Miranda, Portuguesa, Táchira, Yaracuy, Capital District. **Colombia:** Department of Valle del Cauca. **Ecuador:** Provinces ofEsmeraldas,Napo, Pichincha. **Paraguay:** Department of Caaguazú. The treatment of *Leucochrysa (Leucochrysa) pretiosa* by Freitas and Penny (2001: 281) was based on misidentified specimens in the FCAV-UNESP; thus, their distribution records were not included here.

Banks’ (1945: 168) records from Central America (Barro Colorado, Canal Zone; El Cermeno and La Campana, Panama; Cayuga, Volcan [prob. Volcán] Sta. Marta, and Alta Vera Paz, Guatemala; Limon [prob. Limón], Costa Rica) and his records from Colombia (Banks 1944: 31) are all well within the confirmed range of *Leucochrysa (Leucochrysa) pretiosa*. We have seen specimens from most of the areas he reported; however, we have not examined his specimens. The northern-most specimen of *Leucochrysa (Leucochrysa) pretiosa* that we have seen is from Chiapas, Mexico. Valencia Luna et al. (2006: 48) also reported the species from Morelos, Mexico; however, we have not seen specimens to confirm the report. The southern-most record is based on a single specimen, with somewhat obscure data that we interpreted as referring to the Mennonite Colony of Sommerfeld, ca 210 km east of Asunción, Paraguay. This locality is far south of the second southern-most record for the species (north-central Ecuador).

#### Type specimens and rationale for taxonomic changes.

*Allochrysa pretiosa*. Two syntypes from “Inmbo”, Cauca, Colombia, MCZ (one female, one with abdomen missing, examined).

Here, to stabilize the nomenclature of this taxonomically difficult group of lacewings, we recognize the specimen with an abdomen (female) as the **Lectotype [present designation]**.

**Figure 15.**
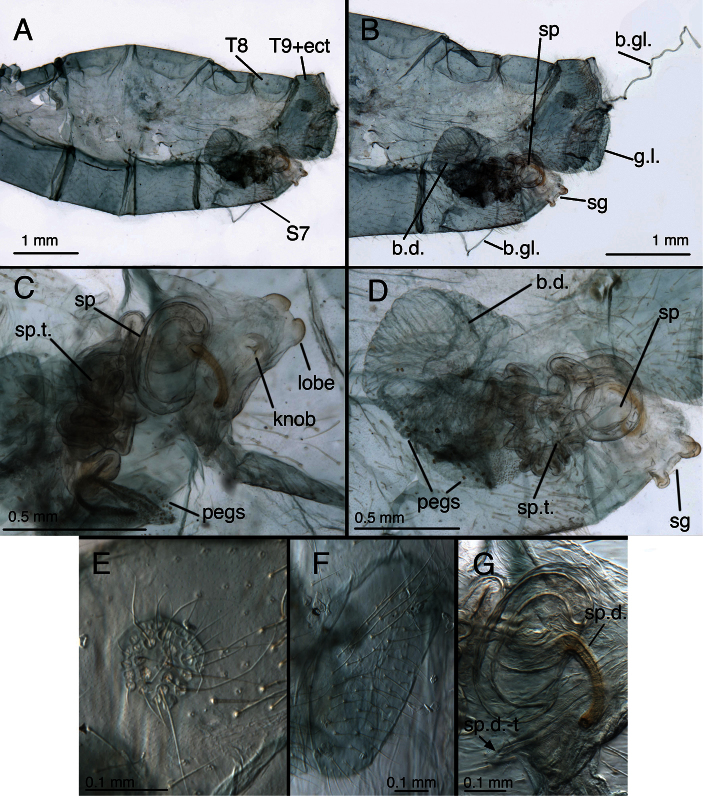
Lectotype, *Allochrysa pretiosa* [= *Leucochrysa (Leucochrysa) pretiosa*], female, MCZ, abdomen and genitalia. **A** Abdominal segments 4–9, lateral **B** Genitalia within terminal segments [Note the bursal glands that extruded from a tear on the right side.] **C** Spermathecal complex and subgenitale, ventral **D** Internal genitalia and subgenitale, lateral **E** Callus cerci **F** Gonapophysis lateralis, lateral **G** Spermathecal duct [The sclerotized section of the duct is brownish; the brushy, membranous section is longer; it extends to the right, bends abruptly to the left, and then ends at the arrow.]. *Abbreviations*: **b.d.** bursal duct **b.gl.** bursal gland **g.l.** gonapophysis lateralis **knob** protruding knob on surface of subgenitale **lobe** distal lobe of subgenitale **pegs** small and large pegs on bursal duct **sg** subgenitale **sp** spermatheca **sp.d.** spermathecal duct **sp.d.-t** brushy terminus of spermathecal duct **sp.t.** tubular section of spermatheca **S7** seventh sternite **T8** eighth tergite **T9+ect** fused ninth tergite and ectoproct.

Its labels read: (1) “Inmbo Cauca / Colombia S. Am.” [Banks’ hand]; (2) “1000 m / Jan” [Banks’ hand]; (3) “Collection / N. Banks”; (4) “Type” [red, Banks’ hand]; (5) “Type / 12005”; (6) “Allochrysa / pretiosa / type / Bks” [white, red border, Banks’ hand]; (7) “LECTOTYPE / *Allochrysa pretiosa* / Banks, 1910 des. / C. A. Tauber 2013” [red]. The specimen is in fairly good condition; the terminalia are in a vial with glycerine. Images, in addition to Fig. 15 here, are in the MCZ Type Database (http://insects.oeb.harvard.edu/MCZ/index.htm). The remaining type is now labelled as a paralectotype.

**Figure 16.**
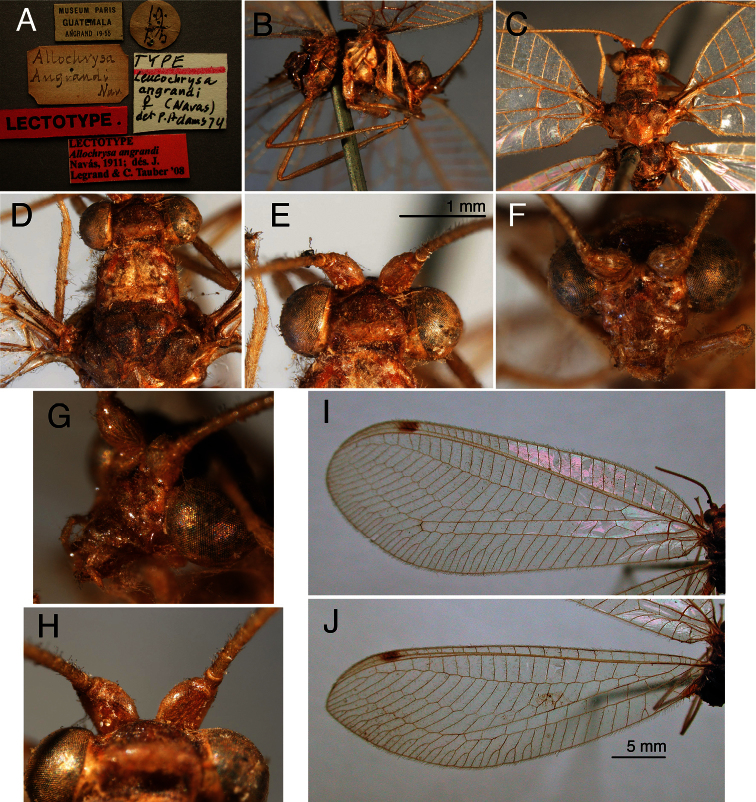
Lectotype, *Allochrysa angrandi*, female, MNHN, external features. **A** Labels **B** Body, lateral **C** Head, thorax, base of wings, dorsal **D** Pronotal, mesonotal markings **E** Head markings, dorsal (slightly obscured) **F** Head, frontal **G** Head, frontolateral **H** Scapes, dorsal **I, J** Forewing and hindwing; scale applies to both wings.

“Cauca” probably refers to the Valle del Cauca, which is in southwestern Colombia. We could not locate an “Inmba” (spelling in original description), “Inmbo” (spelling on lectotype label and by Banks 1944: 31), or “Jumba” (spelling by Navás 1913: 158); we suspect that these names are all misspellings of Yumbo, a town near Cali, Valle del Cauca Department.

*Allochrysa angrandi*. Lectotype, designated by Legrand et al. (2008: 113), MNHN (female, examined).

**Figure 17.**
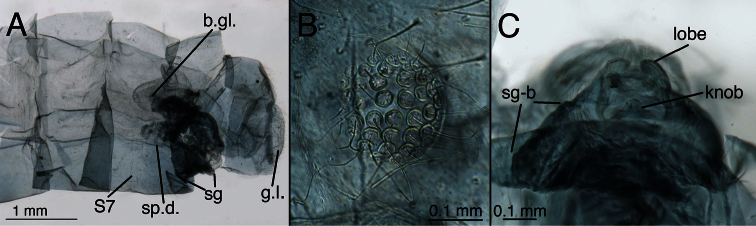
Lectotype, *Allochrysa angrandi*, female, MNHN, external abdomen. **A** Abdominal segments 6–9, lateral (with dorsal tear) **B** callus cerci **C** Subgenitale, ventral. *Abbreviations*: **b.gl.** bursal gland **g.l.** gonapophysis lateralis **knob** protruding knob on surface of subgenitale **lobe** distal lobe of subgenitale **sg** subgenitale **sg-b** broad base of subgenitale **sp.d.** spermathecal duct **S7** seventh sternite.

**Figure 18.**
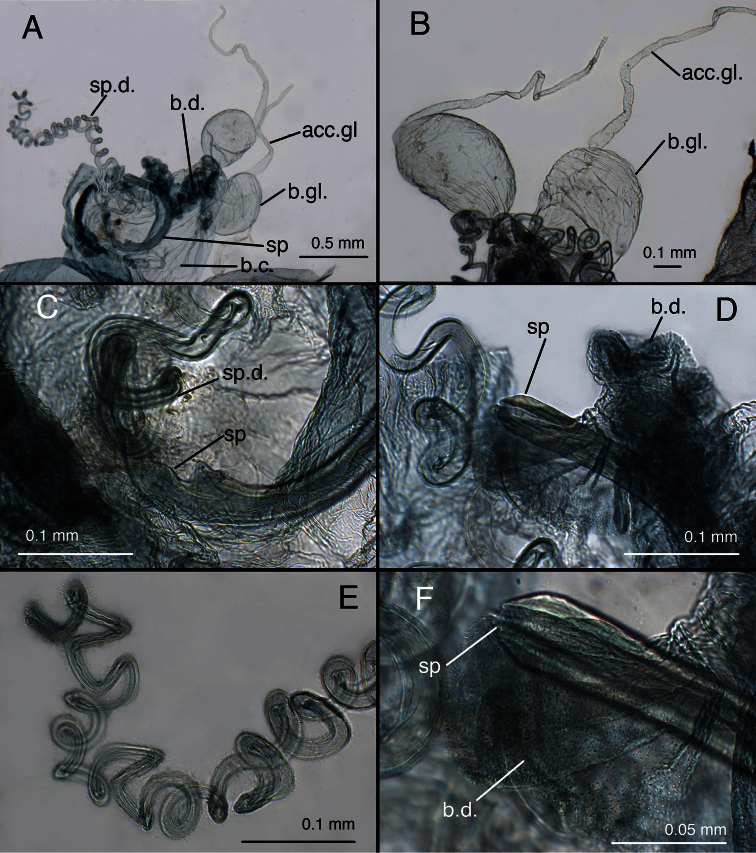
Lectotype, *Allochrysa angrandi*, female, MNHN, genitalia. **A** Bursal and spermathecal complexes [Note the C-shaped spermatheca.] **B** Paired bursal glands with accessory glands **C** Spermatheca at attachment of spermathecal duct **D** Tip of spermatheca at attachment of bursal duct **E** Spermathecal duct with brushy tip **F** Tip of spermatheca, with slit-like opening to setose bursal duct. *Abbreviations*: **acc.gl.** accessory gland of bursal gland **b.c.** bursa copulatrix **b.d.** bursal duct **b.gl.** bursal gland **sp** spermatheca **sp.d.** spermathecal duct.

From his discussion, it appears that Banks (1945: 167) did not see the type of *Allochrysa angrandi*; even so, he suggested that the species was synonymous with *Leucochrysa pretiosa* and, with one exception (Adams 1979: 97), it has been treated as synonymous. Our comparison of the lectotypes of the two species (both females) indicates that they are distinct [compare Fig. 15 with Figs 17–18]. Indeed, the *Allochrysa angrandi* lectotype lacks some characteristics of the *varia*-like species that we have examined. For example, it appears to have red lateral stripes on the prothorax (Figs 16C–E), no suffusion on the forewing crossveins, and the base of the forewing lacks dark markings (Fig. 16I). In addition, the fifth and sixth tergites of the cleared abdomen (Fig. 17) lack the large spots that are typical of *varia*-like species. This characteristic sometimes is not visible in cleared *varia*-like specimens, especially teneral ones, and thus its absence from the *Allochrysa angrandi* type is not definitive for excluding the species from the group. This species will be re-described elsewhere.

*Allochrysa variata*. Lectotype by present designation, BMNH (male, examined).

In his original description, Navás (1912-1913: 315) mentioned two localities; thus, it is clear that he had more than one specimen of this species. We have found only one–the one from Panama (a male in the BMNH). To stabilize the nomenclature of this taxonomically difficult group of lacewings, we designate this specimen as the **Lectotype [present designation]**. Its labels read: (1) “V. De Chiriqui, / 25–1000 ft. / Champion.”; (2) “Godman-Salvin / Collection / 1911–24.”; (3) “Typus” [pink, hand-written, Navás]; (4) “Allochrysa / variata Nav. / Navás S.J. det.” [hand-written, Navás & printed]; (5) “Type / H.T.” [round, with red border]; (6) “LECTOTYPE / *Allochrysa variata* / Navás, 1913 des. / C.A. Tauber 2013” [red]. The specimen is discolored with age, but otherwise in reasonably good condition; the terminalia are in a vial with glycerine.

**Figure 19.**
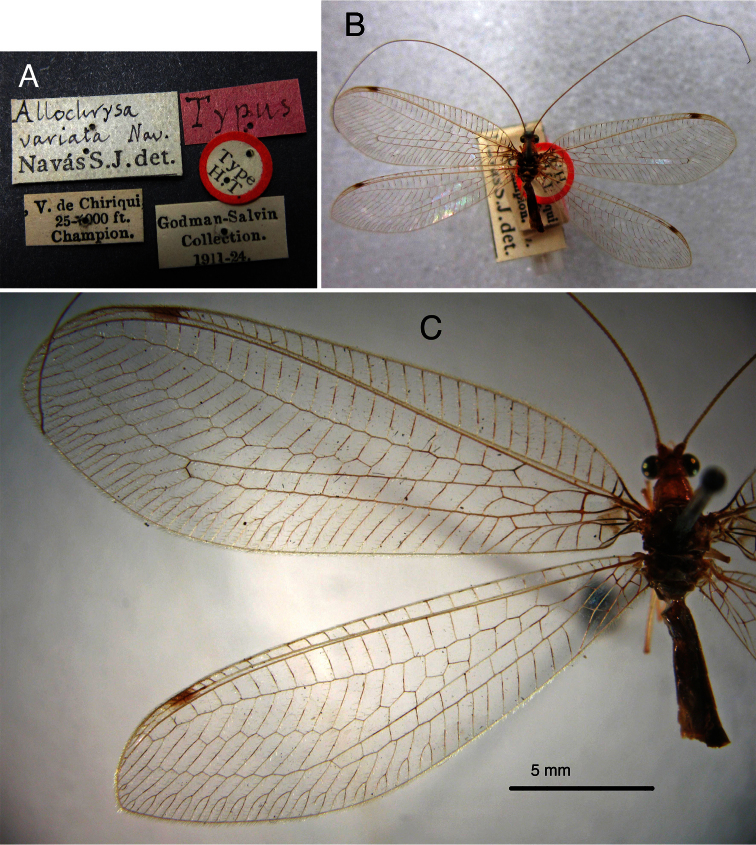
Lectotype, *Allochrysa variata*, male, BMNH, external features. **A** Labels **B** Habitus, dorsal **C** Forewing and hindwing, left.

**Figure 20.**
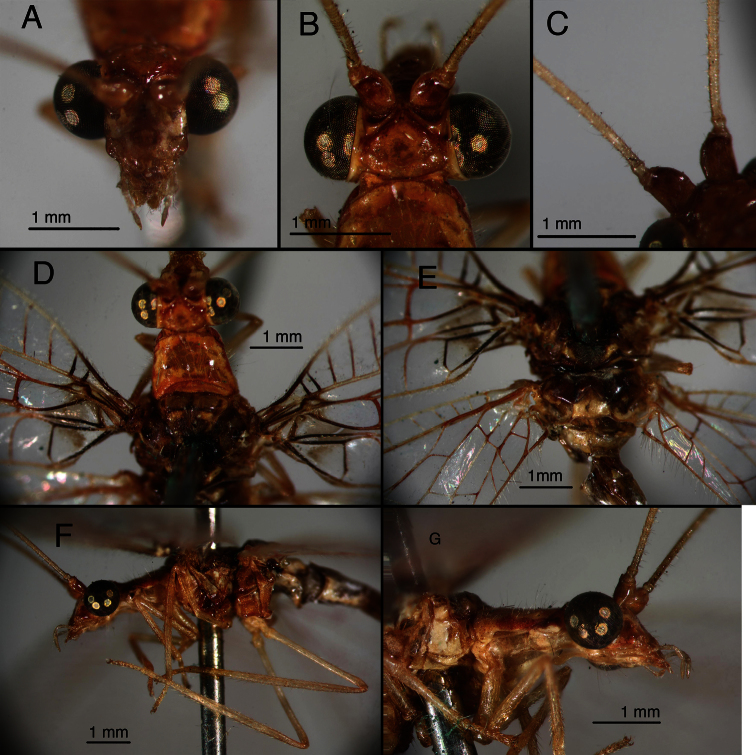
Lectotype, *Allochrysa variata*, male, BMNH, external features. **A** Head, frontal **B** Head, dorsal **C** Scapes, dorsal **D** Pronotum, mesonotum **E** Metanotum **F** Head, thorax, left lateral **G** Head, prothorax, mesothorax, right lateral.

**Figure 21.**
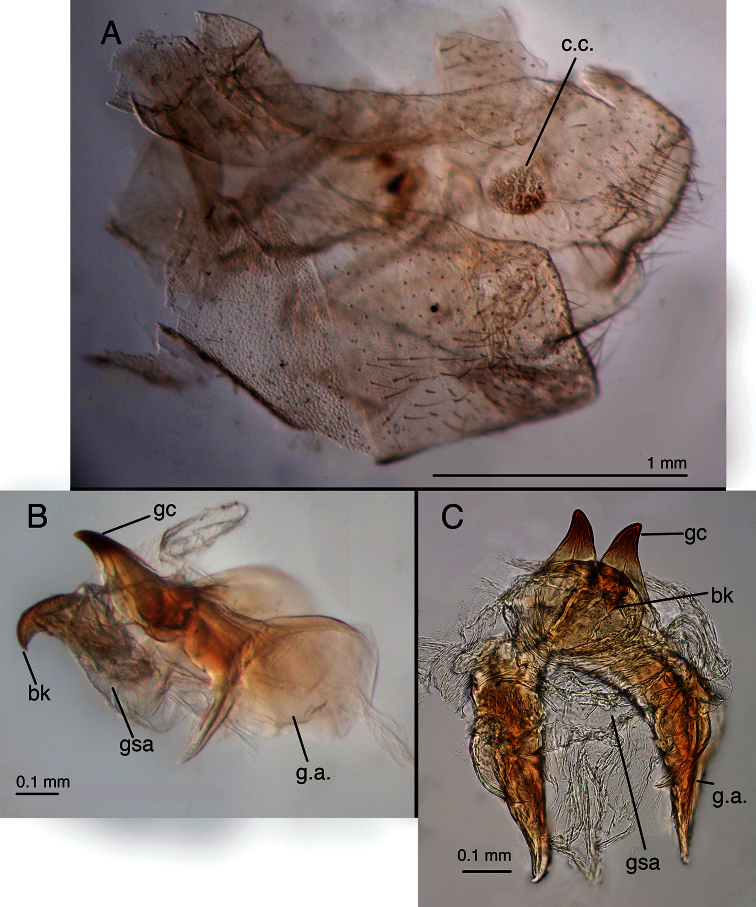
Lectotype, *Allochrysa variata*, male, BMNH, genitalia. **A** Tip of abdomen, lateral (damaged) **B** Gonarcus, lateral **C** Gonarcus, ventral. *Abbreviations*: **bk** beak-like tip of mediuncus **c.c.** callus cerci **gc** gonocornu **gsa** gonosaccus **g.a.** gonarcal apodeme.

**Figure 22.**
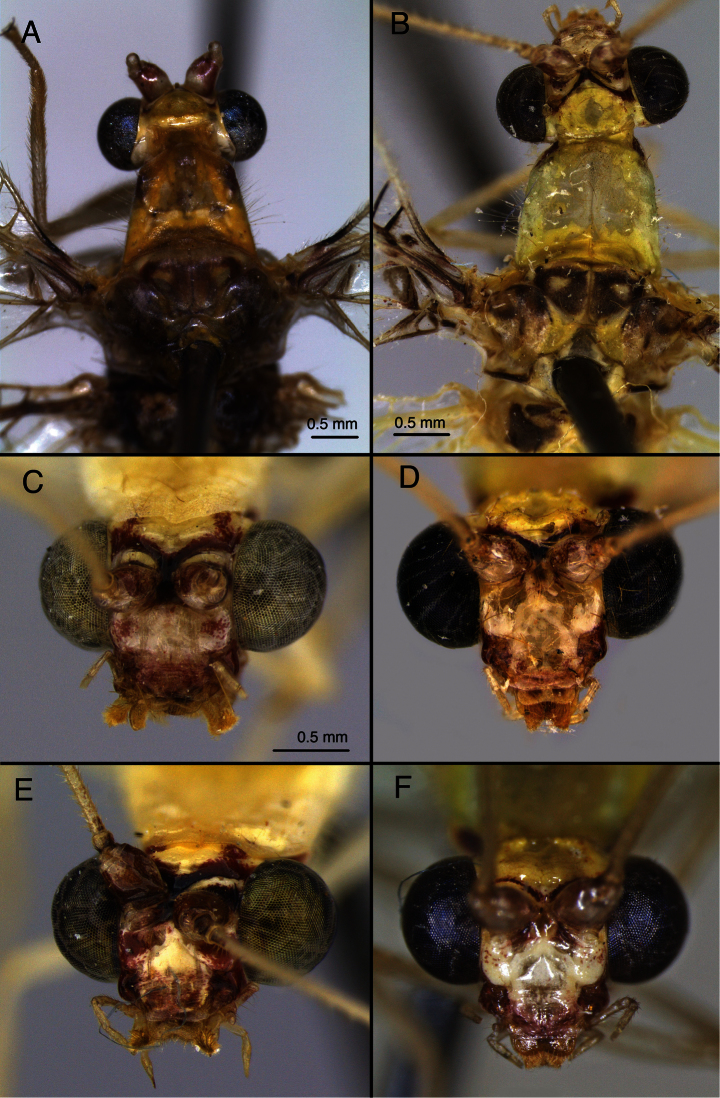
*Leucochrysa (Leucochrysa) pretiosa* variation in head and thoracic markings. **A, B** Head, thorax, dorsal **C–F** head, frontal (A Locality unknown; B, D, F State of Portuguesa, Venezuela; C, E State of Mérida, Venezuela). Scale in **C** applies to **C–F**.

**Figure 23.**
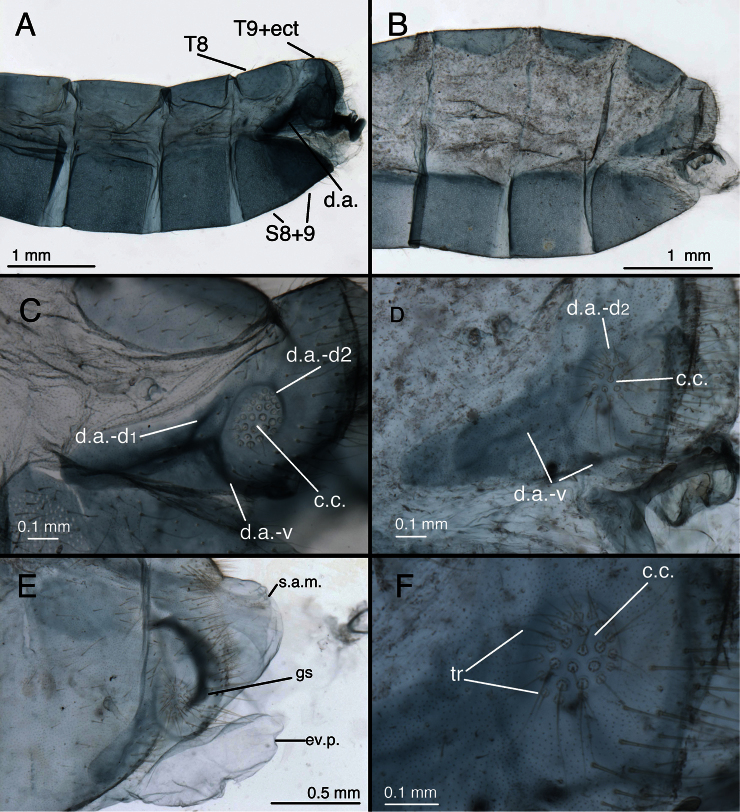
*Leucochrysa (Leucochrysa) pretiosa* male abdominal structures. **A, B** Terminal segments, lateral, demonstrating intraspecific variation in the shape and expansion of the abdomen **C, D** Tergite 9+ectoproct, lateral, illustrating variation in the sclerotization of the dorsal apodeme **E** Abdominal tergite 8 and fused tergite 9+ectoproct, dorsal, slightly tilted to one side **F** Callus cerci (A Province of Esmeraldas, Ecuador B, D, F Trinidad Island, Trinidad & Tobago; C State of Chiapas, Mexico; E Province of Puntarenas, Costa Rica). *Abbreviations*: **c.c.** callus cerci **d.a.** dorsal apodeme of ninth tergite+ectoproct **d.a.-d1, d.a.-d2** first and second dorsal arms of dorsal apodeme **d.a.-v** ventral arm of dorsal apodeme **ev.p.** eversible membranous pouch **gs** gonarcus **s.a.m.** subanal membrane **S8+9** fused eighth and ninth sternites **tr** trichobothria **T8** eighth tergite **T9+ect** fused ninth tergite and ectoproct.

**Figure 24.**
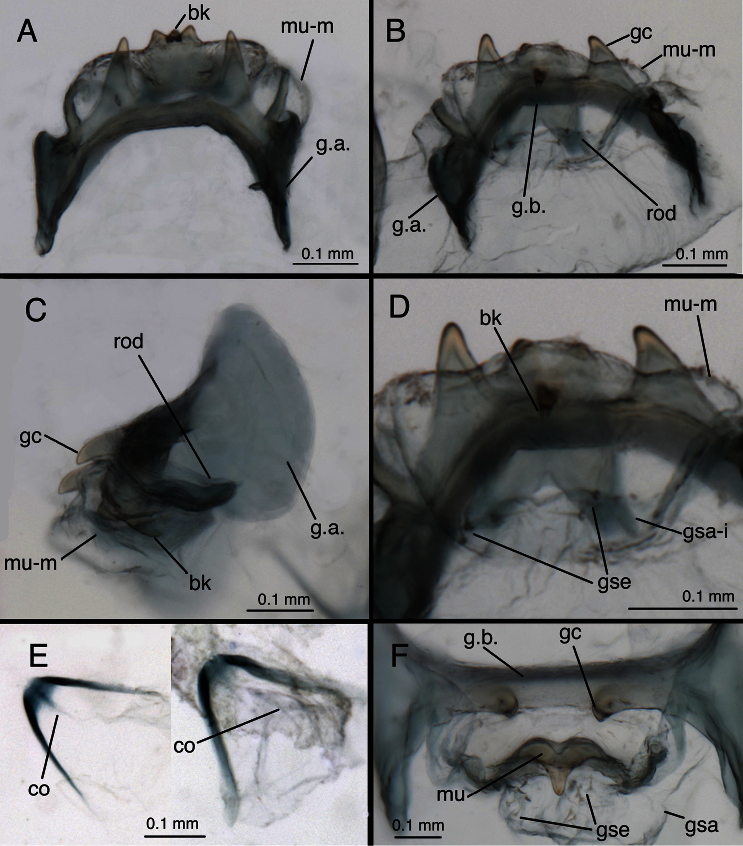
*Leucochrysa (Leucochrysa) pretiosa* male genitalia. **A** Gonarcal complex, dorsal, gonosaccus removed **B** Gonarcus, ventral **C** Gonarcus, lateral, enhanced slightly to increase contrast **D** Mediuncus, ventral **E** Hypandrium internum, illustrating the variation in the shape of the arms and density of connecting membrane **F** Gonarcus and mediuncus, frontal [Note the gonosetae on the inside of the gonosaccus] [A, B, D State of Carabobo, Venezuela; C, E (right), F Trinidad Island, Trinidad & Tobago; E (left) State of Chiapas, Mexico]. *Abbreviations*: **bk** beak-like tip of mediuncus **co** comes **gc** gonocornu **gsa** gonosaccus **gsa-i** interior of gonosaccus **gse** gonosetae **g.a.** gonarcal apodeme **g.b.** gonarcal bridge **mu** mediuncus **mu-m** mediuncal membrane **rod** sclerotized mediuncal rod.

Banks (1945: 167) suggested that *Allochrysa variata* was synonymous with *Leucochrysa pretiosa*, and it was subsequently treated as such. However, our comparison of the *Allochrysa variata* male lectotype with specimens of *Leucochrysa (Leucochrysa) pretiosa* indicates that the two are distinct [compare Figs 19–21 (*variata*) with Figs 22–24 (*pretiosa*)]. This species will be re-described elsewhere.

*Leucochrysa delicata*. Type apparently missing, sex unknown (not examined).

In the original description, Navás stated that he retained the type in his personal collection; it is not there now (Monserrat 1985: 240), nor was it found at the MNHN or the BMNH. Presumably, it is lost (see Adams 1979: 97). Given the collection site of the type (Costa Rica), it is possible that it was either *Leucochrysa (Leucochrysa) angrandi* or *Leucochrysa (Leucochrysa) pretiosa*. Because *Leucochrysa (Leucochrysa) pretiosa* is the more common and therefore most likely of the two, we have no basis for altering Banks’ synonymy; thus it pertains. To help stabilize the taxonomy of this taxonomically difficult group of *Leucochrysa*, we designate a female specimen in the William F. Barr Entomological Collection (UID), University of Idaho, as the Neotype. This specimen is from Turrialba, a locality in the Reventazón River basin of Costa Rica; its labels read: (1) “COSTA RICA. Cart. / Turrialba, CATIE / 26-29 Jun 1986 / Nadeer Youssef”, (2) “NEOTYPE” / *Leucochrysa delicata* / Navás, 1925 des. / Tauber et al. 2013”; (3) “*Leucochrysa (L.)* / *pretiosa* Banks / det. C. A. Tauber 2013”.

*Leucochrysa erminea* Banks. Holotype by original designation, MCZ (sex unknown, abdomen missing, examined). No other type material was found in the MCZ (P. D. Perkins, personal communication).

Banks (1945: 169) did not compare his type of *Leucochrysa erminea* with the *Leucochrysa pretiosa* specimens that he had described many years earlier. He was probably unaware of the variation in body color expressed by this species. We make the synonymy on the basis of external features, notably the wings, which are almost identical on the *Allochrysa pretiosa* and *Leucochrysa erminea* types [See images in MCZ Type Database (http://insects.oeb.harvard.edu/MCZ/index.htm)]. In addition, *Leucochrysa (Leucochrysa) pretiosa* is the most common species of *Leucochrysa (Leucochrysa) varia*-like lacewings in Panama [the type locality of *Leucochrysa (Leucochrysa) erminea*].

#### Diagnosis.

*Leucochrysa (Leucochrysa) pretiosa* expresses the external features listed above for the “*varia*-like” species. In addition, both *Leucochrysa (Leucochrysa) pretiosa* and *Leucochrysa (Leucochrysa) varia* have notable variation in head and thoracic coloration, black shading around the second m-cu crossvein and the distal Psm-Psc crossveins. Although examination of the genitalia is essential for accurate identification of the *varia*-like species, these two species often can be separated externally on the basis of wing characteristics. *Leucochrysa (Leucochrysa) pretiosa* generally has a lighter brown mark on the stigma, a larger, rounded patch of shading around the distal leg of the im1, and slightly more shading around the basal outer gradate veins, second m-cu crossvein, and distal Psm-Psc crossvein than does *Leucochrysa (Leucochrysa) varia*. In general, the distal gradate veins and shaded veins of *Leucochrysa (Leucochrysa) varia* are darker (brown or black) than those of *Leucochrysa (Leucochrysa) pretiosa*.

The males of *Leucochrysa (Leucochrysa) pretiosa* are distinguished by: (i) sternite S3 with microtholi usually present only posterolaterally, rarely anterolaterally or posteromesally [both of which occur in *Leucochrysa (Leucochrysa) varia*]; (ii) much smaller genital structures [gonarcal span of 0.40–0.65 mm; cf., 0.67–0.84 mm in *Leucochrysa (Leucochrysa) varia*]; (iii) gonarcal bridge straight between gonocornua and bent at the interior margin of the gonocornua [not gently curved throughout as in *Leucochrysa (Leucochrysa) varia*]; (iv) basal section of mediuncus broad [extending laterally well beyond gonocornua], leathery, rigid membrane, not a light, flexible membrane as in *Leucochrysa (Leucochrysa) varia*; (v) gonarcus with a pair of slender ventral processes between the lateral apodemes and gonocornua [absent from *Leucochrysa (Leucochrysa) varia*]. Female *Leucochrysa (Leucochrysa) pretiosa* have a very distinctive, bowl-shaped spermatheca, with a relatively short spermathecal duct.

#### Redescription.

*Head* (Fig. 22): 1.7–1.9 mm wide (including eyes). Frons, clypeus white, red suffused, or entirely red; gena pink to dark red; maxillary and labial palpi yellowish. Vertex with central area raised, yellowish to green, with prominent, dark red to dark brown, V-shaped mark along anterior margin; lateral margins with narrow to wide stripes; post-ocular area with red spot. Antenna: scape tinged with red-wine coloration, especially mesally; pedicel yellowish green, inner margin with dark mark; flagellum cream-colored, with amber bristles; inner margin of basal ca three flagellomeres tinged with black; dorsal antennal fossae marked with red laterally.

*Thorax* (Fig. 22):Cervix with red mark laterally. Pronotum 1.1–1.5 mm long, 1.2–1.4 mm wide, yellowish green, unmarked. Mesonotum, metanotum variable, entirely dark brown to yellow with brown marks of various sizes. Posterior margin of mesoscutum with prominent dark brown, transverse stripe; metascutum with small or large brown marks; mesoscutellum, metascutellum yellow to light green, without marks.

*Wings* (Fig. 9A–B) Forewing 18.5–21.8 mm long, 7.2–7.9 mm wide (at widest point); ratio of length: maximum width = 2.51–2.75:1. Costal area moderately broad; tallest costal cell (#8–10) 1.6–1.9 mm tall, 2.4–2.8 times width, 0.2–0.3 times width of wing (midwing). First intramedian cell quadrangular, width (anterior margin) 1.5–2.0 times width (anterior margin) of m3, 1.0–1.2 times length of posterior margin of m3, length of basal vein (= ma, median arculus) 2.0–3.8 times greater than length of distal vein. First radial crossvein distal to origin of radial sector (Rs); radial area (between Radius and Rs) with single row of 15–19 closed cells; tallest cell (#7–10) 1.72–2.80 times taller than wide. No crassate veins; 5–6 b cells (= cells beneath Rs, not including an inner gradate vein). Two series of gradate veins; 8–10 inner gradates, 8–10 outer gradates. Height of fourth gradate cell 3.5–5.5 times width. Seven to eight b’ cells (cells beneath pseudomedia after im2). Three intracubital cells (two closed). Membrane mostly clear except basal area marked with reddish brown, stigma opaque, with large dark brown mark basally, dark clouding around second m-cu crossvein and around distal Psm–Psc crossveins, sometimes with clouding around distal leg of im1 and around crossveins between distal b’ cells. Veins mostly green, except basal costal crossveins, base of Radius, ca three radial crossveins, distal Psm–Psc crossveins, outer gradates, and forks of marginal veins usually entirely black; costal crossveins with black tips; inner gradates, posterior crossveins of distal b’ cells mostly black. Hindwing 16.4–19.4 mm long, 5.5–6.7 mm wide. Two series of gradate veins; 7–10 inner, 7–9 outer; 15–17 radial cells (counted from origin of Radius, not false origin). Five to six b cells (including small b1 cell); seven b’ cells beyond im2; two intracubital cells (one closed). Membrane clear; stigma with pronounced brown mark basally. Veins mostly light green; outer gradates, tips of costal crossveins brown to black.

**Figure 25.**
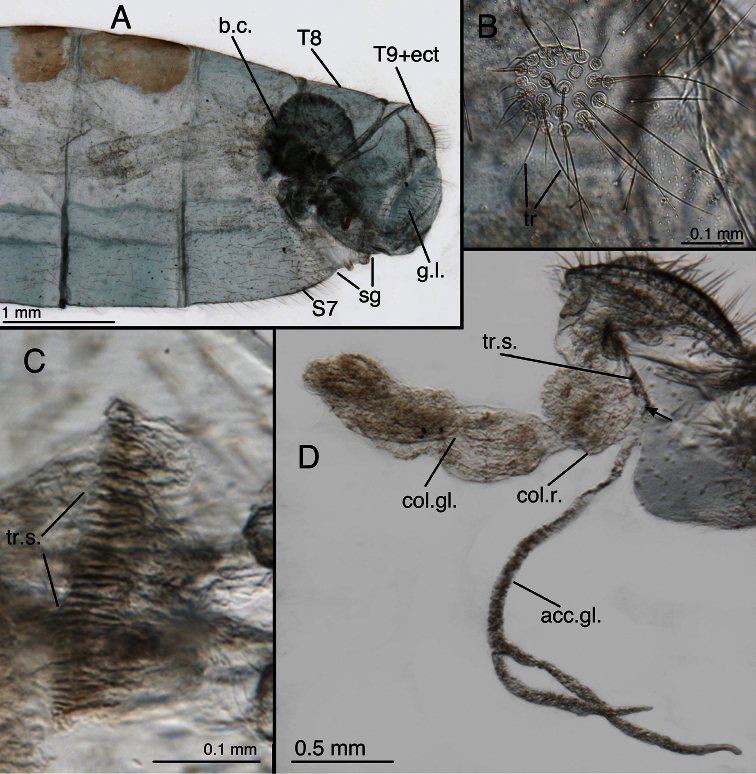
*Leucochrysa (Leucochrysa) pretiosa* female. **A–B** Abdominal structures **C–D** Colleterial complex. **A** Terminal segments, lateral **B** Callus cerci **C** Transverse sclerite **D** Colleterial and accessory glands [The arrow indicates the junction of the duct from the colleterial reservoir with the accessory gland immediately before the structures attach to the shelf of the transverse sclerite.] (A State of Carabobo, Venezuela; B–D Cayo District, Belize). *Abbreviations*: **acc.gl.** bifurcated, granulose accessory gland **b.c.** bursa copulatrix **col.gl.** colleterial gland **col.r.** colleterial reservoir **g.l.** gonapophysis lateralis **sg** subgenitale **S7** seventh sternite **tr** trichobothria **tr.s.** transverse sclerite **T8** eighth tergite **T9+ect** fused ninth tergite and ectoproct.

**Figure 26.**
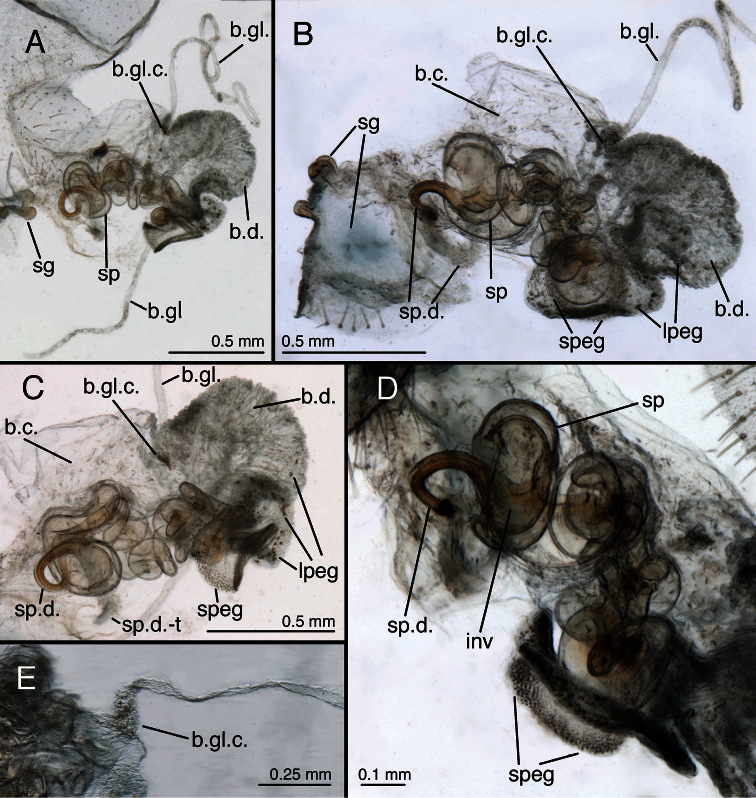
*Leucochrysa (Leucochrysa) pretiosa* female genitalia. **A** Internal genital complex, including bursal glands **B** Bursal and spermathecal complexes, with subgenitale [Note the small pegs at the base of the bursal duct and the larger ones distally.] **C** Bursal and spermathecal complexes **D** Spermathecal complex [Note the large spermathecal invagination that tapers to a narrow tubule in the upper part of the bowl.] **E** Tip of bursa copulatrix with granulose, conical connection with bursal gland (A–D Cayo District, Belize; E State of Carabobo, Venezuela). *Abbreviations*: **b.c.** bursa copulatrix **b.d.** bursal duct **b.gl.** bursal gland **b.gl.c.** connection between bursal gland and bursa copulatrix **inv** spermathecal invagination **lpeg** large peg on distal part of bursal duct **sg** subgenitale **sp** spermatheca **speg** small peg on basal part of bursal duct **sp.d.** spermathecal duct **sp.d.-t** brushy terminus of spermathecal duct.

*Abdomen* (Figs 15, 23–26): Tergites with mostly short, slender setae throughout, sternites with longer, slender setae; microsetae dense; pleural region with setae small, very sparse, microsetae very small. Tergites narrow, roughly rectangular, with rounded or irregular margins. Spiracles oval externally; atria not enlarged. Sternites S2–3 longer than wide; S5–7 more square-shaped (lateral view); distal segments (beyond A4) expanded, height of pleural region greater than height of sternites [integument of cleared specimens soft, floppy, easily damaged]. Coloration: mostly green, with yellow mesally. Tergites T5, T6 with large black spots, bordered by red; callus cerci white; setae, trichobothria golden.

*Male*. Height and length of S6 ca equal, S7 height ca 1.1–1.2 times length (lateral view). Microtholi dense on S4–S8, usually sparse, only present distolaterally on S3 (rarely, a few microtholi along posteromesal edge), absent from S1–2, S9. Callus cerci slightly oval (ca 1.2–1.3× taller than wide), diameter 0.16–0.23 mm, with 28–38 trichobothria of variable length. T9+ectoproct soft, lightly sclerotized, rounded posterodorsally, truncate to rounded distally, broadly fused mesally, midline with small distal cleft; ventral section rounded, tapering proximally, extending proximally only to suture line between fused S8 and S9 (dorsal margin); dorsal apodeme lightly to moderately sclerotized, straight, with two dorsal forks before callus cerci, curving along ventral margin of ectoproct to terminus; first branch of dorsal apodeme extending posterodorsally to level midway up callus cerci; second branch extending around proximal, dorsal and posterodistal margins of callus cerci. S8+9 fused, with trace of suture dorsally, with clear intersegmental demarcation throughout; S8 1.6–2.0× taller than long, less than one-half (0.36–0.45×) length of S8+9; S8+9 (lateral view) with proximal margin slightly convex, dorsal surface gradually curving ventrally over 3/4ths distance from proximal margin, then curving steeply to terminus; terminus without knob or gonocristae; membrane above terminus with pair of large, eversible, membranous pouches. Gonarcus well sclerotized, arcuate, total span: 0.40–0.65 mm; bridge broad, straight mesally, curved abruptly at interior margin of gonocornua, dorsoventrally flattened, distance between apodemes 0.30–0.36 mm; lateral apodeme bell-shaped, broader ventrally than dorsally (0.32–0.36 mm wide, 0.18–0.19 mm tall); gonocornua extending from lateral edge of gonarcal bridge, basally stout, tapering to narrow, rounded apex, length 0.08–0.13 mm; distance between inner bases of gonocornua 0.08–0.13 mm, distance between tips 0.11–0.16 mm. Gonarcus, between lateral apodeme and gonocornu, with moderately sclerotized, elongate posteroventral processus with membrane attached. Mediuncus with basal section consisting of broad, leathery, membranous plate extending from distal margin of gonarcal bridge, recurving below gonarcal bridge; distal section of mediuncus consisting of heavily sclerotized, flat, broadly V-shaped plate, below and well separated from gonarcus; distal plate with mesal beak extending ventrally, deep mesal trough dorsally, between rounded, lateral ridges that extend above beak; membrane below gonarcus forming deep pouch with two fields of three to six stout gonosetae on chalazae; fields of gonosetae on surface of membrane facing mediuncus. Hypandrium internum: arm 0.20–0.24 mm long, distal span between arms 0.17–0.24 mm.

*Female*. Height of S6 ca 0.62–0.70 times length, S7 height ca 0.51–0.57 times length. Callus cerci round, diameter ca 0.15 mm, with 24–28 trichobothria. Tergite T8 roughly quadrate (lateral view) with rounded corners, similar in depth to T6. T9+ectoproct elongate, slanting anteriorly; ventral margin convex, extending slightly below level of gonapophyses laterales. Dorsal margin of S7 straight, tapering abruptly distally; terminus unmodified, with terminal (posteroventral) setae slightly more numerous, longer than in other areas. Gonapophysis lateralis rounded throughout, ca 0.53–0.58 times height of T9+ectoproct; inner membranous surface slightly expandable, with vertical patch of small, delicate setae arising from slightly swollen membrane. Colleterial complex consisting of membranous gland connected to colleterial reservoir via broad duct, and elongate, ribbon-like accessory gland, both opening to exterior via narrow ducts above transverse sclerite; colleterial gland elongate, robust; colleterial reservoir shorter, robust; accessory gland narrow, elongate, granular, forked distally; transverse sclerite broad, flat to slightly convex, with broad, robust teeth. Spermatheca bowl-shaped basally (0.3–0.4 mm width x 0.2 mm height), with invagination tubular, extending around wall of spermathecal bowl; distal, tubular section of spermatheca broad, convoluted with ca 10 complete loops; spermathecal duct short, ca 0.8 mm including membranous, brushy, distal section, with single short, well sclerotized loop basally, longer, lightly sclerotized, brushy loop distally. Bursa copulatrix saccular, with heavily textured surface near bursal duct, becoming smoother posteriorly; bursal duct membranous, broad, flat, folded, spinose basally (near spermathecal velum), becoming broader, granulose, with patches of small, then large, robust pegs distally (near base of bursa); pair of elongate, tubular bursal glands attached to base of each side of bursa via enlarged, granular, conical bases. Subgenitale with smooth (unfolded), rounded surface, with bilobed projection dorsally, ca two times wider distally than at base, midsection with prominent lobe extending outward at ca 90° angle to subgenitale surface.

#### Larvae.

Unknown.

**Biology.** Unknown.

#### Variation.

*Leucochrysa (Leucochrysa) pretiosa* expresses considerable variation in head, mesothoracic and metathoracic coloration, as well as wing size, shape and degree of suffusion on various veins. The range of variation in several of these traits is shown on Figs 9 and 22. As in *Leucochrysa (Leucochrysa) varia*, the expansion of abdominal segments 4–9 and the degree of sclerotization of the apodemes and sclerites varies considerably among specimens (see Figs 23, 25). This variation may be related in part to age and/or maturation; however, both male and female specimens with large, expanded (apparently mature) abdomens often had very soft and delicate integuments that tore easily during dissection. Thus, a delicate integument may not necessarily be associated with a teneral status.

#### Material examined

**(in addition to the types listed above). MEXICO. *Chiapas*:** San Jerónimo, Tacaná, 6.ix.1970, T. W. Taylow Colln. (1♂, LACM). **BELIZE. *Belize*:** 1 mi. N. Sibun R., 14 mi. N. Belize City, in cork forest, 5.vii.1973, Y. Sedman (1♂,CAS); ***Cayo*:** 5 mi. N. San Ignacio, 12–13.xii.1988, F. D. Parker (1♀, USU). **COSTA RICA. *Puntarenas*:** Golfito, 23.vii.1957, Truxel & Menke (1♀, LACM); Golfito-United Fruit Co., 2.vii.1976, Malaise trap 8A-5P, M. Wasbauer (1♂, SDCM). **NICARAGUA. *No department***: Eden [14°0'N, 84°26'W] (locality not confirmed), Th. W. Bouchelle (1♂, PNAS, det. by N. Banks). **PANAMA. *Canal Zone*:** Pipeline Road, 22.iii.1982, W. J. Hanson (1♀, USU); Barro Colorado Island, 14.ii.1955, C. W. Rettenmeyer (1♀, SEM); idem, 9.i.1929, C. H. Curran (1♀, AMNH). **TRINIDAD & TOBAGO. *Trinidad Island*:** St Andrew Parish, Brigand Hill, 21.vii.1979, L. Sorkin (1♀, AMNH); Arima Valley, 800–1200 ft, 5–15.ii.1965, J. G. Rozen (1♀, AMNH); Arima Valley, 4.ii.1953, J. G. Rozen (1♀, AMNH); “Naracas” [= “Maracas”] Valley, 18.v–10.vi.1957, ROM party (1♀, ROM); Simla Res. Sta., 2–15.vi.1961, Hanson, Clemons (3♂, 1♀, USU). **VENEZUELA. *Amazonas*:** Cerro de la Neblina Basecamp, 140 m, 0°50'N, 66°9'44"W, 13–20.ii.1984, D. Davis & T. McCabe (1♀, USNM); idem, 3.ii.1985, on low foilage, rainforest trail, W. E. Steiner (1♀, USNM); ***Aragua*:** Parque Nacional Henri Pittier (formerly Parque Nacional Rancho Grande), 1100 m, 7.iii.1959, C. J. Rosales (1♀, MIZA); idem, 1–5.i.1966, S. S. & W. D. Duckworth (1♀, USNM); idem, 11–15.i.1966, S. S. & W. D. Duckworth (1♀, USNM); 1 km S. Rancho Grande, 5.ii.1976, C. M. & O. S. Flint, Jr. (1♂, USNM); Ocumare [1123099N - 0547148W], 100 m, 20.ii.2008, F. Sosa, F. Díaz & R. Zúñiga (1♂, UCOB); ***Capital District*:** Caracas, R. M. Bartleman (1♀, USNM); ***Carabobo*:** San Esteban, Las Quiguas, 185 m, 5–8.x.1974, J. Salcedo, R. Dietz & J. L. García (1♂, 1♀, MIZA); Yuma, 13.v.1980, F. Fernández Y & A. Chacón (without abdomen, MIZA); nr Canoabo, 850 m, 24.i.1983, O. S. Flint, Jr. (5♂, 6♀, USNM); ***Falcón*:** Sierra de San Luis, Valle de Acarite, 980 m, 15.vii.1983, J. Lattke (1♂, MIZA); ***Lara*:** Parque Nacional Yacambú, El Blanquito, 1100 m, 19.i.2011, F. Sosa & J. Torres (2♀, UCOB); Santa Rosa de la Fila, Finca Dos Aguas, 1300 m, 5–8.iii.2011, H. Chavez & A. Chavez (1♀, UCOB); Parque Nacional Terepaima, 1100 m, 12.i.2012, F. Sosa & D. R. R. Fernandes (1♀, UCOB); ***Mérida*:** El Pedregal, 200 m, 22.i.2009, F. Sosa & F. Díaz (2♀, UCOB); idem, 25.i.2009, F. Sosa & F. Díaz (1♂, 3♀, UCOB); ***Miranda*:** Parque Nacional Guatopo, km 24 N. Altagracia de Orituco, 640 m, 5–9.v.1975, malaise trap, J. Salcedo & R. Dietz (1♂, 2 without abdomen, MIZA); ***Portuguesa*:** Araure, Finca Barra de Oro [9°36'N, 69°19W], 310 m, 11.i.2008, F. Sosa & A. López (1♂, 1♀, UCOB); La Estación, Los Borbollones [9°22'N, 69°28'W], 310 m, 26.xii.2007, F. Sosa (1♀, UCOB); ***Táchira*:** Paramillo, UNET, 1050 m, 23.vii.2007, F. Sosa & F. Díaz (1♂, UCOB); ***Yaracuy*:** San Felipe, Hacienda Guaquira [10°17'N, 68°39 W], 100 m, 14.ii. 2010, F. Sosa & J. Torres (2♂, 2♀, UCOB); idem, 13.i.2012, F. Sosa & D.R.R Fernandes (2♂, 1♀, UCOB). **ECUADOR. *Esmeraldas*:** Parr. San Mateo, 4.v.1956 (1♂, CAS, bought from F. H. Walz, PAA); ***Napo*:** Misahualli nr. Tena, 26.viii–2.ix.2000, S. & P. Keller (1♂, USU). ***Pichincha*:** E. Sto. Domingo, 8–16.v.1988, Hanson & Bohart (1♀, USU). **PARAGUAY. *No district*:** Summerfield, 7.x.1965, rec MAZ, A. C. Allyn, Acc. 1969–20 (1♀, FMNH). [The locality in Paraguay probably is the Sommerfeld (Mennonite) Colony in Caaguazú Department.]

## Supplementary Material

XML Treatment for Leucochrysa (Leucochrysa) varia

XML Treatment for Leucochrysa (Leucochrysa) pretiosa
